# A Metastable Oxygen Redox Cathode for Lithium‐Ion Batteries

**DOI:** 10.1002/anie.202422789

**Published:** 2025-02-11

**Authors:** Yanfang Wang, Cheng Li, Yingzhi Li, Raquel de Benito, Jacob Williams, Joshua M. Stratford, Zhiqiang Li, Chun Zeng, Ning Qin, Hongzhi Wang, Yulin Cao, Dominic Gardner, Wilgner Lima da Silva, Sahil Tippireddy, Qingmeng Gan, Fangchang Zhang, Wen Luo, Joshua W. Makepeace, Ke‐Jin Zhou, Kaili Zhang, Fucai Zhang, Phoebe K. Allan, Zhouguang Lu

**Affiliations:** ^1^ Department of Materials Science and Engineering Southern University of Science and Technology Shenzhen 518055 China; ^2^ School of Chemistry University of Birmingham Edgbaston, Birmingham B15 2TT UK; ^3^ Department of Electronic and Electrical Engineering Southern University of Science and Technology Shenzhen 518055 China; ^4^ The Faraday Institution Harwell Campus Didcot UK; ^5^ Diamond Light Source Harwell Campus Didcot UK; ^6^ Department of Mechanical Engineering City University of Hong Kong Kowloon Hong Kong China

**Keywords:** Metastable, Anion Redox, Oxygen Activity, Layered Cathode, Lithium-ion Batteries

## Abstract

Simultaneously harnessing cation and anion redox activities in the cathode is crucial for the development of high energy‐density lithium‐ion batteries. However, achieving long‐term stability for both mechanisms remains a significant challenge due to pronounced anisotropic volume changes at low lithium content, unfavorable cation migration, and oxygen loss. Here, we demonstrate exceptionally stable cation and anion redox behavior in a metastable, cobalt‐free layered oxide, Li_0.693_[Li_0.153_Ni_0.190_Mn_0.657_]O_2_ (LLNMO). After 50 cycles at 50 mA/g (~0.2 C), the cathode retains 97.4 % of its initial capacity (222.4 mAh/g) with negligible voltage decay. This remarkable stability is attributed to its metastable O6‐type structure (R‐3m symmetry) with unique local geometry. The face‐sharing connectivity between lithium layers and alternating transition metal (TM) layers effectively suppresses TM migration‐induced voltage decay during anion redox. Additionally, the structure balances interlayer cation/cation and anion/anion repulsions, resulting in minimal expansion and contraction during de‐/lithiation (<2.3 % along the *c*‐axis) and excellent structural reversibility. These findings highlight that layered oxides with a metastable framework are promising cathode candidates for next‐generation ultra‐high‐energy lithium‐ion batteries.

## Graphical abstract

A cobalt‐free Li_0.693_[Li_0.153_Ni_0.190_Mn_0.657_]O_2_ (LLNMO) exhibits outstanding electrochemistry by harnessing both cation and anion redox activities. It features a metastable structure with both edge‐sharing (E) and face‐sharing (F) local geometries, distinct from conventional O3‐type structures. The face‐sharing local configuration provides enhanced stability and flexibility to modulate the interlayer interactions during de‐/lithiation.

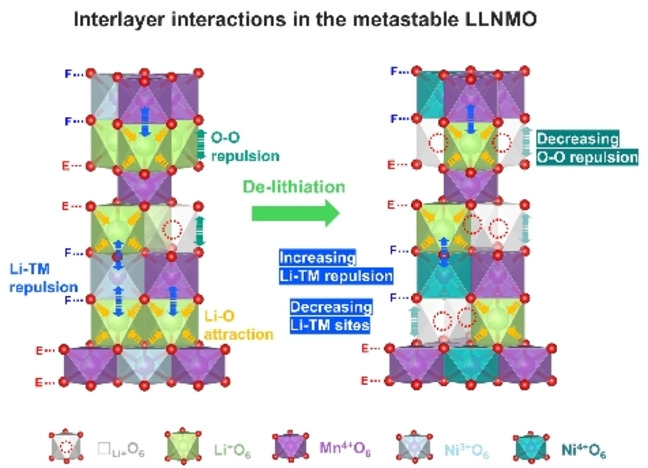

## Introduction

Developing high‐capacity cathode materials which exploit both cation and anion redox is widely acknowledged as the forefront strategy for the development of ultra‐high‐energy‐density lithium‐ion batteries (LIBs).[^1^, ^2^] However, for both charge compensation mechanisms, achieving long‐term stability is extremely challenging. In the case of cation redox in layered lithium transition metal oxides (such as Ni‐rich Li[Ni_x_Mn_y_Co_1‐x–y_]O_2_, x>0.6), high levels lithium removal can trigger structural instability with the loss of O_2_.[^3^, ^4^] A large reduction in the *c*‐parameter is commonly observed at low lithium content, with a degradation of cell performance due to intergranular cracking from repeated two‐phase reactions,^[5]^ or high interfacial lattice strain between the bulk structure and the reconstructed surface layer.[^6^, ^7^] Meanwhile, harnessing the very high capacities theoretically afforded by anion redox reactions (ARRs) is more challenging still. For example, in the traditional O3‐type cathode materials (where “O” designates the alkali metal in octahedral sites and 3 designates the number of TMO_2_ layers in the repeating unit),^[8]^ triggering ARRs in Li[Li_x_Ni_y_Mn_z_Co_1‐x–y–z_]O_2_ (Li‐rich NMC) can deliver capacities higher than 250 mAh/g, but ARRs jeopardize their cycling stability, leading to rapid capacity fading and voltage decay.[^9^, ^10^, ^11^]

Intensive studies have been conducted to elucidate the mechanisms of oxygen redox, revealing the pathway that lattice oxygen (O^2−^) can be oxidized to O−O dimers (O_2_^n−^, 0<n<4) and potentially to molecular O_2_ across 3d, 4d, and 5d TM based cathode materials.[^12^, ^13^, ^14^] Considering that the O−O dimerization requires the cleavage of TM−O bonds, the de‐coordinated TM ions are prone to migrate either within TM layers or into Li layers, both of which are thermodynamically favorable in deeply de‐lithiated states.^[15]^ The instability of cathode materials with oxygen redox activity largely originates from irreversible TM migrations. Specifically, the intralayer TM migration can destroy ordered local patterns and induce voltage hysteresis,^[16]^ while the interlayer TM migration leads to the layered‐to‐spinel phase transformation, which is thought to be one origin of voltage decay.^[17]^ Furthermore, TM migration favors TM−O de‐coordination, promotes irreversible oxygen loss from the surface, and can produce electrochemically inactive O_2_ trapped in the bulk, thereby resulting in capacity fading.^[18]^ From this perspective, mitigating TM migrations is essential to stabilize ARRs in high‐capacity cathode materials.

Substantial effort has been devoted to suppressing TM migrations in O3‐type cathode materials. For example, approaches to tuning TM/Li arrangement, e. g., tailoring ribbon‐like superstructures (as opposed to hexagonal) and building totally disordered local patterns, have been demonstrated to be effective in mitigating the intralayer TM/Li mixing.[^19^, ^20^] However, as to the interlayer TM migration, although various methods, e. g., local structure design, cation/ anion doping and surface coating, have demonstrated improved cycling properties for O3‐type cathode materials,[^21^, ^22^] preventing the cumulative spinel phase formation is still a challenge for long‐term cycling. From the structural point of view, the difficulty stems from the edge‐sharing local geometry in O3‐type structures, which favors the interlayer TM migration. In other words, once TM ions have entered intermediary tetrahedral sites in Li layers, their migration into adjacent octahedral sites to form TM_Li_−V_TM_ anti‐site defects is inevitable and irreversible.^[23]^ Recently, building O2‐type cathodes with face‐sharing local structures has been demonstrated to be feasible in suppressing the irreversible TM migration.^[24]^ Specifically, although both O3‐type and O2‐type structures are similarly built up via the layer‐by‐layer stacking of LiO_2_ and TMO_2_ slabs, LiO_6_ octahedra only share edges with adjacent TMO_6_ octahedra in the former, whereas they share both edges and faces with TMO_6_ octahedra in the latter.^[25]^ Therefore, in O2‐type structures, there are higher energy penalties for TM ions to migrate from intermediate tetrahedral sites to octahedral sites, owing to the large electrostatic repulsion between face‐sharing TM ions.^[26]^

Herein, we report a metastable cobalt‐free cathode material Li_0.693_[Li_0.153_Ni_0.190_Mn_0.657_]O_2_ (named LLNMO), formed via ion‐exchange from a sodium‐containing P2‐type (where “P” designates the alkali metal in prismatic sites) precursor to form a O2+T2 (where “T” designates the alkali metal in tetrahedral sites) mixture followed by heat treatment at a moderate temperature, which causes a phase transition from the O2+T2 mixture to a metastable structure showing rhombohedral symmetry (R‐3m, O6‐type). The metastable LLNMO exhibits exceptional cycling stability utilizing both reversible cation and anion redox reactions, which we propose originates from the unique layer stacking arrangement. Inherited from O2‐type structures, its face‐sharing geometry on one side of the lithium layer suppresses TM migration whilst also providing an additional repulsive Li^+^−TM^4+^ interaction at the face‐sharing sites. This decreases during charging which counteracts the increasing O^2−^−O^2−^ repulsion as screening lithium is removed, serving to minimize lattice expansion/contraction.

## Results and Discussion

### Preparation and Structural Characterizations

**Figure 1:**
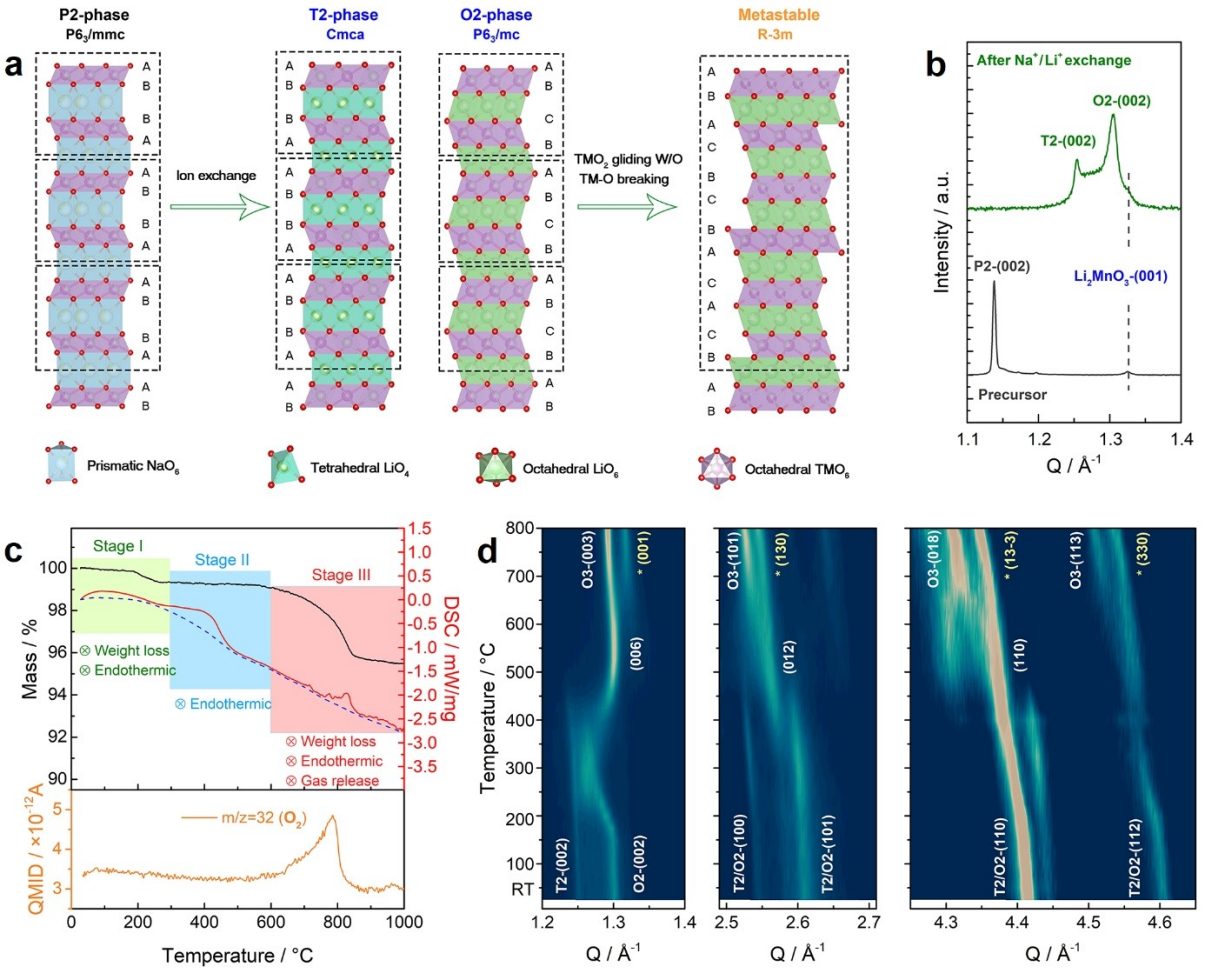
Structural evolutions after Na^+^/Li^+^ ion exchange and during the subsequent thermal treatment. (a) Schematic illustration of preparaing the metastable LLNMO. (b) Synchrotron XRD patterns (λ=0.6887 Å) of the P2‐precursor and the O2+T2 mixture. (c) TGA‐MS results of the O2+T2 mixture. On the top: the black solid line represents the TG curve; the red solid line represents the DSC curve, and the blue dashed line represents the baseline. On the bottom: quasi multiple ion detection (QMID) ion current curve collected via mass spectrometry for m/z=32 (O_2_). (d) Contour maps of in situ VT‐XRD patterns (λ=1.54 Å) for the O2+T2 mixture. Peaks labeled by asterisks can be indexed to a spinel phase (Fd‐3m symmetry).

The metastable O6‐type LLNMO was prepared via a facile Na^+^/Li^+^ ion‐exchange from a P2‐type precursor followed by thermal treatment at moderate temperatures, as illustrated in Figure 1a. Firstly, the P2‐type Na_0.745_[Li_0.153_Ni_0.194_Mn_0.626_]O_2_ was synthesized through a traditional solid reaction method, whose composition and structure were determined by the inductively coupled plasma‐mass spectrometry (ICP‐MS) test and Rietveld refinement of its synchrotron X‐ray diffraction (XRD) patterns, respectively (details in Figure S1 and Table S1, S2). In the P2‐type structure (P6_3_/mmc symmetry), the orientation of the TMO_2_ slab differs from one layer to its neighboring layers, forming an ABBA stacking pattern along the *c*‐axis.^[27]^ Upon Na^+^/Li^+^ ion‐exchange, the P2‐type precursor can transform via layer translations into either T2‐type (Cmca symmetry, ABBA stacking pattern) or O2‐type (P6_3_/mc symmetry, ABCB stacking pattern) structures (Figure 1b), in which the alternating orientation of the TMO_2_ slabs is retained.[^28^, ^29^, ^30^] The O2+T2 mixture shows poor crystallinity, as featured by broad XRD peaks and nanodomains observed in high angle annular dark‐field‐scanning transition electron microscopy (HAADF‐STEM) images (Figure S2).

Thermal stability of the ion‐exchanged O2+T2 mixture was investigated via conducting thermal gravimetric analysis‐mass spectrometry (TGA‐MS) and in situ varied temperature (VT)‐XRD tests (Figure 1c, 1d). Upon heating up to 300 °C, the O2+T2 mixture lost 0.7 % weight because of the evaporation of adsorbed water, while all diffraction peaks shift to lower Q presumably due to thermal expansion. Upon further heating up to 600 °C, although no weight loss was observed, an endothermic peak appeared at around 400 °C in the differential scanning calorimetry (DSC) curve. Meanwhile, both the O2‐ and T2‐type structures transform to a structure with R‐3m symmetry. Such an intermediate phase is clearly metastable: it decomposes into two phases—a layered phase (also R‐3m symmetry) and a spinel phase (Fd‐3m symmetry)—at temperatures above 600 °C as indicated by the mass loss (release of O_2_) and the split of the main XRD peak (Figure S3). The metastable LLNMO used in this work was prepared via heating the ion exchanged sample at 500 °C for 12 h, which clearly has crystal structures different from the O2+T2 mixture (Figure S4).

To study the structure of the metastable LLNMO, Rietveld refinements of its synchrotron XRD patterns were performed. Considering that it has a R‐3m symmetry, a typical O3‐type structure (*a*=*b*=2.8624(1) Å, *c*=14.2881(7) Å) was firstly used, and the fitting is good (R_w_=6.42 %, G.O.F.=1.65, reduced χ^2^=2.72, Figure S5 and Table S3). However, in the stable O3‐type structure, all TMO_2_ slabs have the same orientation and only share edges with adjacent LiO_2_ slabs, in which TM ions can migrate via the edge‐sharing plane to the octahedral sites in the Li layers, favoring the detrimental layered‐to‐spinel phase transformation (Figure S6a). As mentioned above, TMO_2_ slabs in either T2‐ or O2‐type structure display alternating orientations (i. e., the two neighboring TMO_2_ slabs have different orientations), in which the irreverisble interlayer TM migration is suppressed due to the face‐sharing local geometry (Figure S6b).^[15]^ Therefore, forming a stable O3‐type structure from an O2+T2 mixture requires the rearrangement of the relative TMO_2_ slab orientations, the cleavage of the TM−O bonds, and the release of O_2_, which is clearly untriggered during moderate thermal treatment. In fact, solely via the gliding of TMO_2_ slabs and without breaking any TM−O bonds, an O6‐type structure with R‐3m symmetry can form as well, which is arguably an intergrowth between O2‐ and O2’‐type structures (Figure S7).^[27]^

**Figure 2:**
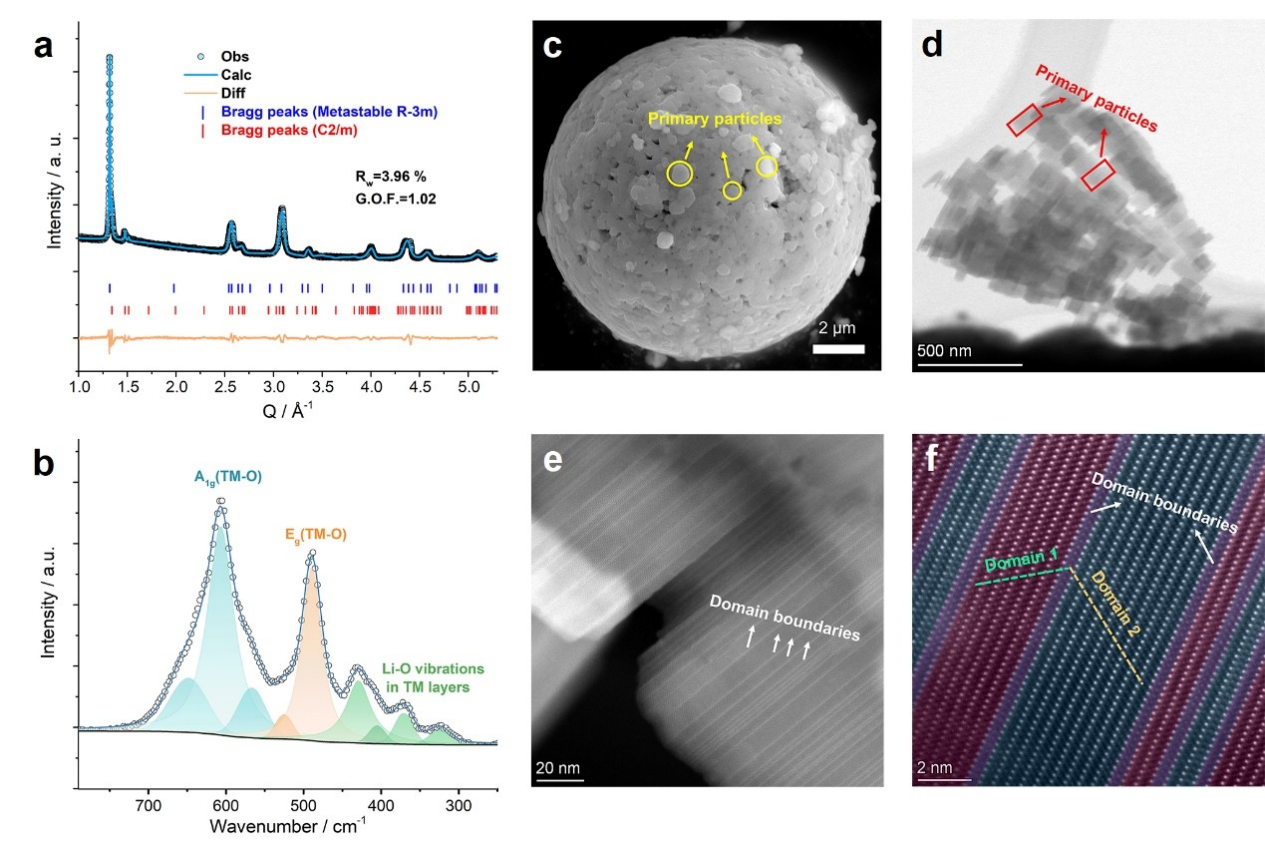
Structural characterization of LLNMO. (a) Synchrotron XRD patterns (λ=0.6887 Å) refined with a metastable structure (R‐3m symmetry, 95.4 wt%) and the impurity of Li_2_MnO_3_ (C2/m symmetry, 4.6 wt%). (b) Raman spectra. E_g_ and A_1g_ refers to symmetric deformation and symmetric stretching of TM−O bonding, respectively. (c) SEM image and (d–f) HAADF‐STEM images of LLNMO. In (f), regions coloured in dull red and light blue represent nanodomains with different orientations, while the violet ones represent their boundaries.

A better refinement was achieved with such a structure with cell parameters of *a*=*b*=2.8618(7) Å and *c*=28.5728(9) Å (R_w_=5.86 %, G.O.F.=1.51, reduced χ^2^=2.27, Figure S8 and Table S4). As shown in Figure 2a and Table S5, the value of G.O.F. was further reduced to 1.02 after adding a small impurity of Li_2_MnO_3_ (4.6 wt%), which already exists in the P2‐type precursor. Both the superstructural peaks at around Q=1.47 Å^−1^ and Raman‐active Li−O vibrations in the region of 300–450 cm^−1^ (Figure 2b) indicate the existence of Li^+^ ions in TM layers.^[31]^ Such results are well supported by time‐of‐flight (TOF) neutron diffraction data (Figure S9).

Accordingly, the metastable LLNMO has R‐3m symmetry and ideally, in the long range, exhibits an ABACBCBACACB stacking pattern along the *c*‐axis (O6‐type). As for the local geometry (Figure S10), the LiO_2_ slabs share edges with adjacent TMO_2_ slabs on one side and share faces with others on the other sides (denoted as EF‐type), which are same with the O2‐type structure. Specially, for the TMO_2_ slabs, they are either exclusively edge‐sharing (EE‐type) or face‐sharing (FF‐type) with adjacent LiO_2_ slabs.

Morphologically, the metastable LLNMO has plate‐like primary paticles and well‐crystalline layered structures (Figure 2c–2f, S11). Due to its unique synthetic approach, plenty of domain boundaries (DBs) form in parallel to the basal planes of the primary particles. Considering that planar strains usually cause structural degradations in layered materials,^[32]^ such ordered DBs can potentially act as buffer zones to even out the strain accumulation and enhance structural stability.

### Electrochemical Performance

**Figure 3:**
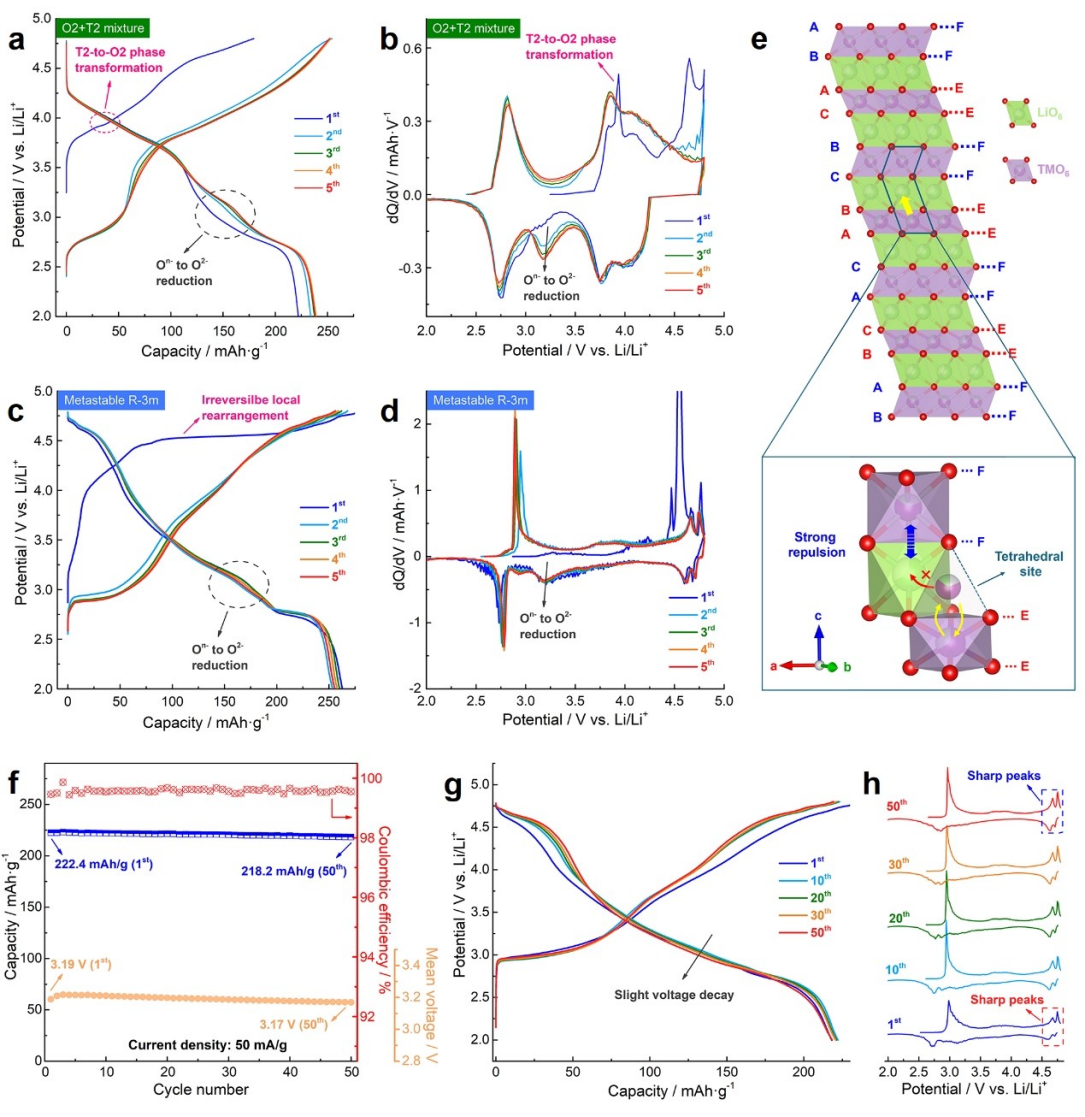
Electrochemical properties. (a) 1^st^–5^th^ voltage profiles, and (b) corresponding dQ/dV curves of the O2+T2 mixture. (c) 1^st^–5^th^ voltage profiles, and (d) corresponding dQ/dV curves of the metastable LLNMO. The current density is 10 mA/g, and the potential window is 2.0–4.8 V vs Li/Li^+^. (e) Crystal structures (top) and TM migration paths (bottom) in the metastable R‐3m phase. The projection vector is [010]. E and F denote edge‐sharing and face‐sharing planes, respectively. (f) Cycling performance of the LLNMO cathode at the current density of 50 mA/g within the potential window of 2.0–4.8 V vs Li/Li^+^ (after 5 cycles at 10 mA/g for activation). (g) Voltage profiles and (h) corresponding dQ/dV curves in the 1^st^, 10^th^, 20^th^, 30^th^ and 50^th^ cycles.

The metastable O6‐type LLNMO exhibits electrochemical properties that are distinct from previously reported O2‐/O3‐type cathodes and from the as‐prepared O2+T2 mixture (Figure 3).[^21^, ^22^] As shown in Figure 3a, 3b, the O2+T2 mixture is electrochemically active within both a low potential region (i. e., 2.5–3.0 V vs Li/Li^+^) and a high potential region (i. e., 3.5–4.5 V vs Li/Li^+^), relating to redox reactions of Mn and Ni, respectively.^[33]^ During the initial de‐lithiation, the sharp dQ/dV peak at around 3.9 V vs Li/Li^+^ can be assigned to an irreversible T2‐to‐O2 phase transformation.^[34]^ Afterward, both broad redox peaks and sloping voltage profiles imply that the formed O2‐type structure undergoes single‐phase reactions in following cycles. In addition, the extra reduction peak at around 3.2 V vs Li/Li^+^ implies oxygen redox reactions (i. e., O^n−^ to O^2−^ reduction).^[35]^

As for the metastable LLNMO cathode (Figure 3c, 3d), upon the initial charging to 4.6 V, it presents classical voltage profiles with a slope before the flat plateau at around 4.5 V, which can be attributed to irreversible local rearrangement induced by oxygen redox activities.^[31]^ Uniquely, two additional plateaus appear in the high potential region (4.6–4.8 V), which are highly reversible in discharge and in the following cycles. Besides, the sharp dQ/dV peaks suggest reversible phase transformation processes. In discharge, peaks at around 3.2 V in the dQ/dV curves also imply oxygen reduction. In the low potential region (2.0–3.0 V), flat plateaus at around 2.8 V suggest another reversible biphasic process, as supported by sharp dQ/dV peaks. Such biphasic processes are in close relationship with its unique local structures, vide infra.

Analogous to the O2‐type structure, the unfavorable interlayer TM migration can be suppressed by the face‐sharing local geometry in the metastable LLNMO. As illustrated in Figure 3e, upon de‐lithiation, TM ions in the TM layers can migrate to the tetrahedral sites in the Li layers via passing through the edge‐sharing planes. However, the octahedral sites in the Li layers also have shared faces with the adjacent TMO_2_ slabs, meaning that the subsequent intralayer migration to the octahedral sites in the Li layers is prohibited by the strong electrostatic repulsion between the face‐sharing sites.

Upon cycling, the metastable LLNMO shows an initial capacity of 222.4 mAh/g at 50 mA/g (~0.2 C) and a 97.4 % capacity retention after 50 cycles in a flurinated electrolyte (Figure 3f–3h), while the mean voltage drops slightly from 3.19 V to 3.17 V (0.4 mV per cycle). In addition, the corresponding dQ/dV peaks are still sharp after 50 cycles, demonstrating excellent structural stability. Such performances are competitive with many state‐of‐the‐art cobalt‐free O2‐ and O3‐type cathodes with oxygen redox activities (Table S6).[^15^, ^36^, ^37^, ^38^, ^39^] Considering that no surface modification was employed, the bared surface of the metastable LLNMO cathode can react with the electrolyte severely due to its significant oxygen redox activities. Therefore, the metastable LLNMO electrode shows inferior cycling stability in an ordinary electrolyte (1M LiPF_6_ in EC/DMC (1 : 1 in volume)), though the initial electrochemical properties are identical in both electrolytes (Figure S12). Such performance degradation originates from the continuous electrolyte consumption and the lack of robust cathode‐electrolyte interphase (CEI),^[40]^ as supported by electrochemical impedance spectroscopy (EIS) and X‐ray photoelectron spectroscopy (XPS) results (Figure S13, S14).

### Redox Mechanisms

**Figure 4:**
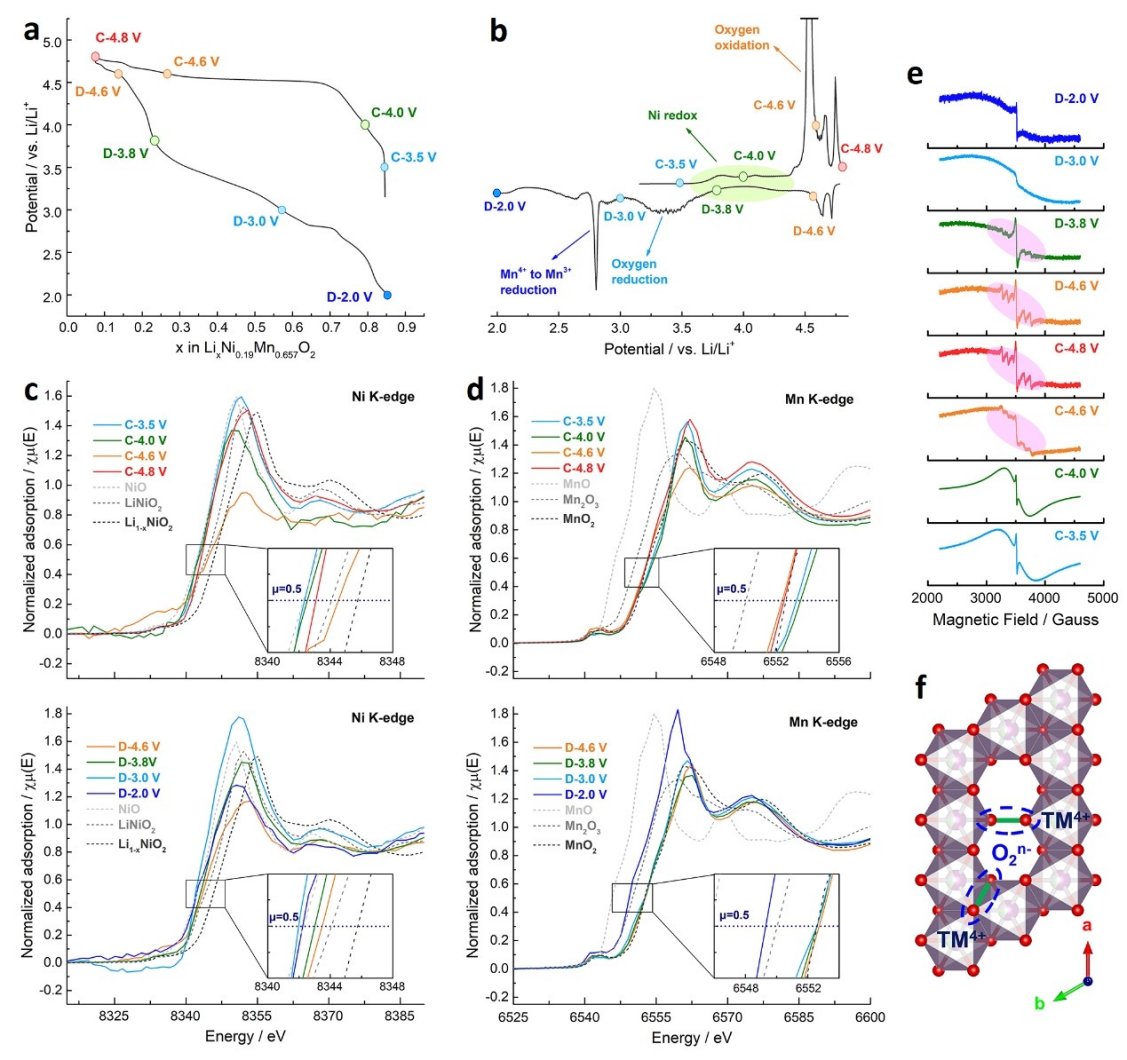
Characterization of redox mechanisms in LLNMO. (a) Voltage profiles and (b) corresponding dQ/dV curves in the 1^st^ cycle. The current density is 5 mA/g, and the potential window is 2.0–4.8 V vs Li/Li^+^. The points where LLNMO electrodes were collected for ex situ tests are marked by coloured circles. (c) Ni K‐edge and (d) Mn K‐edge XAS results at different states of charge and discharge. All spectra are normalized, and the insets show enlarged regions at around μ=0.5. (e) The ex situ EPR results of LLNMO electrodes collected at different states of charge and discharge. All spectra are normalized based on mass. (f) The illustration of local patterns in the de‐lithiated LLNMO with Li vacancies and O−O dimers (O_2_^n−^, 0≤*n*<4).

Having Li^+^ ions in TM layers leads to the formation of Li‐O−Li or Li−O‐vacancy configuration, which usually favors anion redox activities. In addition, both the flat plateau in the 1^st^ charging profile and the sharp dQ/dV peak at around 4.5 V vs. Li/Li^+^ imply local rearrangements induced by oxygen redox (Figure 4a and 4b).^[41]^ Therefore, to uncover the underlying redox mechanisms in the metastable LLNMO, ex situ X‐ray absorption spectroscopy (XAS), XPS, electron paramagnetic resonance (EPR), and high‐resolution resonant inelastic X‐ray scatting (HR‐RIXS) tests were performed at various states of charge and discharge during the initial cycle.

In pristine LLNMO, Mn is mainly in the valence state of 4+, while the valence state of Ni is between 2+ and 3+ (Figure S15). Upon the initial charging (C‐3.5 V→C‐4.0 V and C‐4.6 V; where C refers to charge), the Ni K‐edge shifts towards higher energy, confirming the Ni oxidation in the initial voltage slope (Figure 4c). Meanwhile, the binding energy of lattice oxygen (O^2−^) in the O 1s XPS spectra stays at 529.8 eV after charging to C‐4.0 V, while further charging over the plateau (C‐4.0 V→C‐4.6 V) produces the emerging shoulder at a higher energy (530.5 eV), indicating the formation of dimerized O_2_^n−^ (0≤*n*<4) species caused by oxygen oxidation (Figure S16a).^[17]^

It is noteworthy that both Ni and Mn K‐edges slightly shift to lower energy in the deeply charged state (C‐4.8 V) due to a reductive coupling behavior with oxygen redox activities (Figure 4d), as previously reported.^[42]^ On discharge (D‐4.6 V→D‐3.8 V and D‐3.0 V; where D refers to discharge), Mn K‐edge is stable, while the Ni K‐edge shifts to lower energy, indicating the Ni^3+^/Ni^4+^ to Ni^2+^ reduction. The XPS features from dimerized O_2_^n−^ (0≤*n*<4) species disappear during D‐3.8 V→D‐3.0 V (Figure S16b), indicating reversible oxygen reduction. Upon further discharging (D‐3.0 V→D‐2.0 V), the features resulting from the Ni K‐edge and lattice oxygen barely change, while the Mn K‐edge shifts to lower energy, manifesting Mn^4+^ to Mn^3+^ reduction.

In EPR spectra (Figure 4e), at the beginning of the initial charge (C‐3.5 V), broad peaks are observed because the metastable LLNMO is paramagnetic at room temperature, as shown by the magnetic susceptibility test (Figure S17). When charged to C‐4.0 V, the broad peaks maintain upon solely Ni oxidation. However, triggering anion redox reactions (C‐4.0 V→C‐4.6 V and C‐4.8 V) weakens those peaks and produces six‐fold hyperfine patterns (marked by pink ellipses), implying the existence of unique local environments. Firstly, oxygen oxidation induces anisotropic distortions of TMO_6_ octahedra and disturbs the magnetic exchange interaction between TM and oxygen ions.^[43]^ In addition, removing Li from TM layers creates vacancies and favors the formation of O−O dimers (O_2_^n−^, 0≤*n*<4), which break the percolating network mediated by oxygen.

Therefore, as illustrated in Figure 4f, such six‐fold hyperfine patterns reveal the TM^4+^−O_2_^n−^ (0≤*n*<4) coupling in the vacancy‐mediated structure.^[44]^ On discharge, those patterns remain at D‐3.8 V and finally disappear at D‐3.0 V, implying that reducing the dimerized O_2_^n−^ (0≤*n*<4) back to lattice oxygen (O^2−^) is polarized,^[33]^ consistent with XPS results. The broad peaks restore at D‐3.0 V and D‐2.0 V with weaker signals, presumably resulting from irreversible local rearrangement and the Mn^4+^ to Mn^3+^ reduction, respectively.

**Figure 5:**
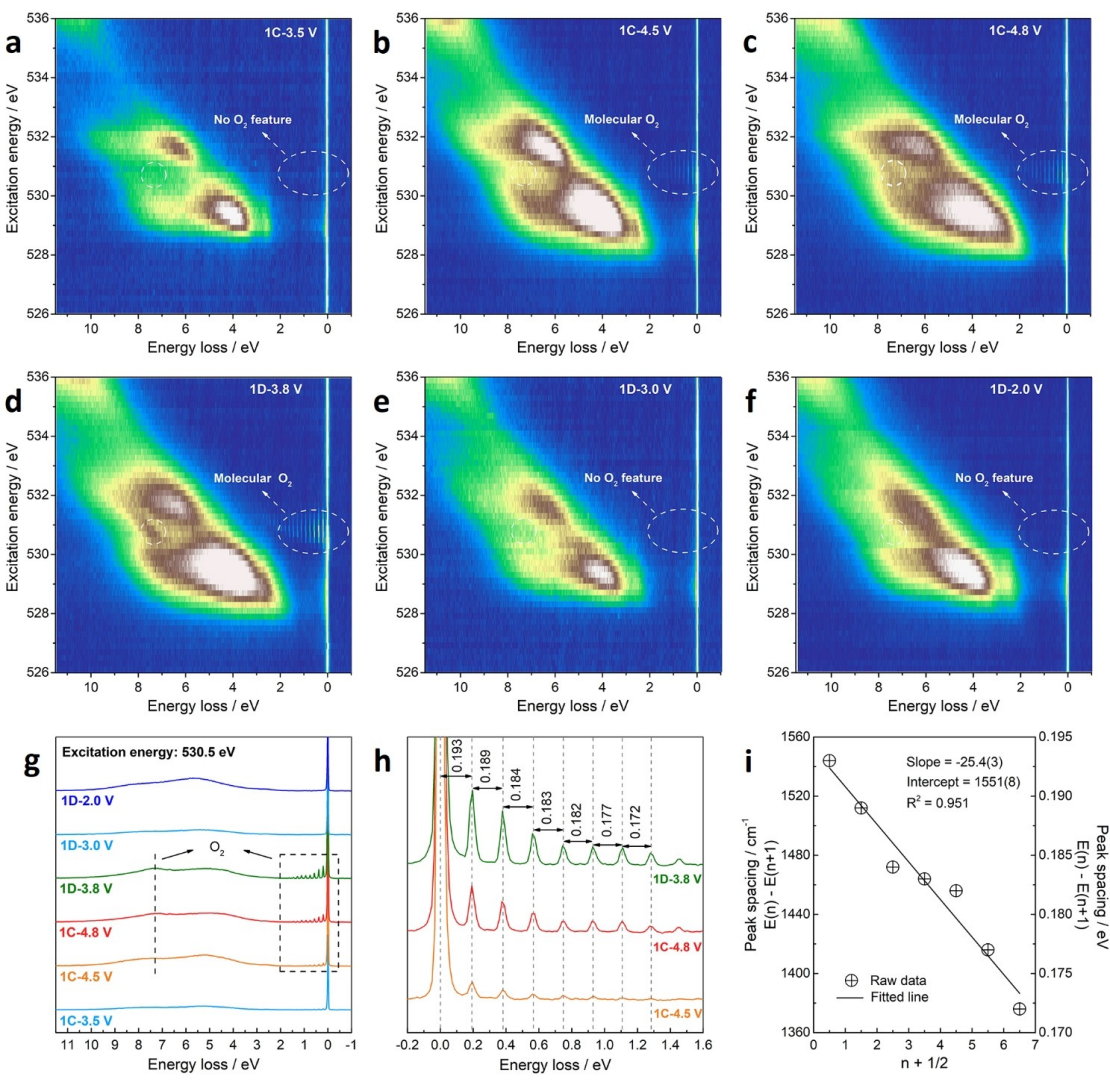
Characterization of bulk oxygen redox activities in LLNMO. (a–f) RIXS maps of O K‐edge at C‐3.5 V, C‐4.5 V, C‐4.8 V, D‐3.8 V, D‐3.0 V, and D‐2.0 V in the initial cycle. The molecular O_2_‐related features (i. e., a progression of energy loss peaks between 0–2 eV and a broad peak at 7.5 eV energy loss) emerge at the beginning of the oxygen redox process (C‐4.5 V) and disappear after discharge, manifesting the reversible formation and reductions of O_2_ molecules. (g) HR‐RIXS line scans obtained at the resonance energy for the vibrational peaks arising from O_2_ (530.5 eV). (h) Peak spacings between the vibrational peaks. (i) Birge‐Sponer plot showing the linear decrease in peak spacing characteristic of an anharmonic oscillating diatomic, wherein n is vibrational quantum number and y‐intercept value indicates a fundamental vibrational frequency close to that of molecular O_2_ (~1556 cm^−1^).

The bulk oxygen redox activities were further proved by O K‐edge HR‐RIXS maps (Figure 5a–5f), as evidenced by new characteristic emission features at around 530.5 eV.[^13^, ^14^] The resulting RIXS line scans at this excitation energy display two main features, i. e., a broad peak at 7.5 eV energy loss and a series of sharp peaks between the energy loss of 0–2 eV (Figure 5g, 5h). The peaks (i. e., the fine structures of the elastic peak) arise from transition between different vibrational energy levels of an O−O diatomic, whose fundamental vibrational frequency is 1551 cm^−1^ according to the Birge–Sponer plot (Figure 5i), closely matching that of molecular O_2_ (~1556 cm^−1^).^[45]^ The molecular O_2_‐related RIXS features still exist at D‐3.8 V, revealing that the hysteretic oxygen reduction is mainly involved in the D‐3.8 V→D‐3.0 V process, as evidenced by the disappearing O K‐edge RIXS features and XPS peaks at D‐3.0 V. Such reversible formation and reduction of O_2_ molecules were also observed in the 2^nd^ cycle (Figure S18).

### Structural Evolutions

**Figure 6:**
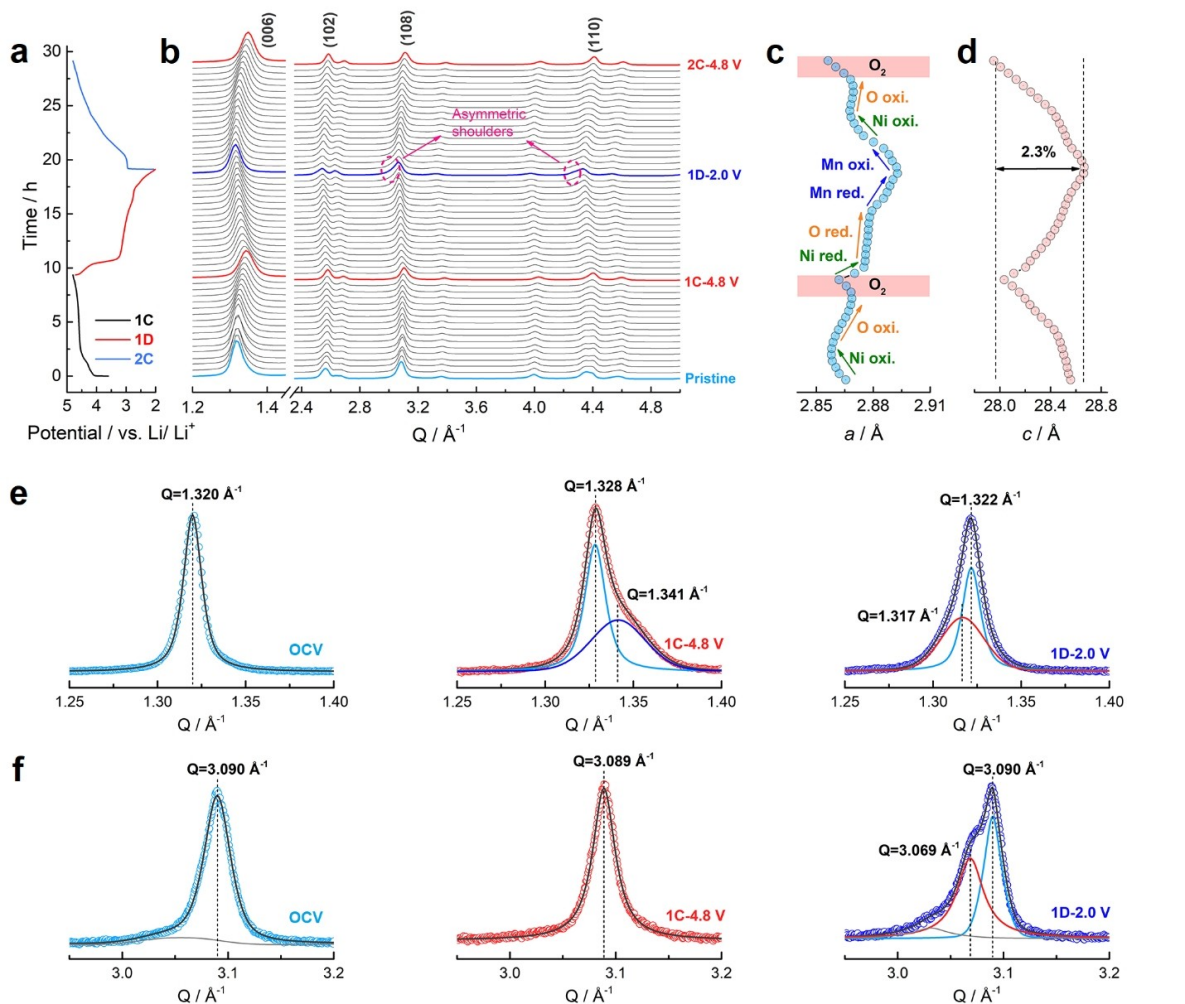
Structural evolution of LLNMO upon de‐/lithiation. (a) Voltage profiles, (b) operando synchrotron XRD patterns (λ=0.16167 Å), and (c, d) calculated cell parameters. In (a), the current density is 20 mA/g, and the potential window is 2.0–4.8 V vs Li/Li^+^. Ex situ XRD patterns (Cu Kα, λ=1.54 Å) showing (e) fitted (006) peaks and (f) fitted (108) peaks of the LLNMO electrodes at OCV (left), 1 C–4.8 V (middle) and 1 D–2.0 V (right).

To reveal structural evolutions of the metastable LLNMO during de‐/lithiation, operando synchrotron XRD tests were performed in the 1^st^ cycle and the 2^nd^ charge (Figure 6a, 6b). Generally, it undergoes reversible structural changes. The lattice parameters were calculated according to the positions of the (006) and (108) peaks (Figure 6c, 6d).

Lattice parameter *a* is sensitive to the TM−O bond length within the TMO_2_ slabs and usually changes during cation redox activities. Upon the 1^st^ charge, *a* decreases during the initial slope (OCV→1 C–4.5 V) due to the shortening of the Ni−O bond length induced by Ni oxidation. In the following oxygen oxidation process (1 C–4.5 V→1 C–4.8 V), *a* increases, which might result from the irreversible local structure rearrangement. On discharge, *a* increases upon Ni reduction, barely changes upon oxygen reduction and further increases upon Mn reduction. The 2^nd^ charge follows the reverse change of parameter *a*. It is noteworthy that, in the deeply charged states, the decrease of *a* manifests lattice shrinkage presumably induced by the formation of molecular O_2_. Such TMO_6_ distortions within the basal plane (*ab*‐plane) were also captured by ex situ and operando Raman results (Figure S19).

Unlike *a*, for layered structures, lattice parameter *c* is related to the interlayer interactions between different TMO_2_ slabs. In the metastable O6‐type LLNMO, *c* changes in ways different from that of the typical O3‐type cathodes, though both have R‐3m symmetry. Such performance originates from its face‐sharing local structures, which *de facto* manipulate the interlayer interactions. In typical O3‐type structures, two competing forces (i. e., the attractive Li^+^−O^2−^ interaction and the repulsive O^2−^−O^2−^ interaction) determine the interlayer spacing, as illustrated in Figure S20a. Upon initial de‐lithiation (cation oxidation), removing Li^+^ reduces the attraction between Li^+^ and O^2−^. Hence, the interlayer spacing increases due to the stronger electrostatic repulsion between the O layers.^[46]^ Upon further de‐lithiation (oxygen oxidation), the interlayer spacing gradually decreases because removing electrons from O^2−^ ions weakens their repulsions.

However, in the metastable O6‐type structure observed in LLNMO, the Li^+^−TM^n+^ repulsion is strong at the face‐sharing sites, which acts as an additional force and affects the overall interlayer interactions (Figure S20b). Specifically, upon the initial de‐lithiation (cation oxidation), the O^2−^−O^2−^ repulsion increases due to the diminishing screening effect of Li^+^. On the other hand, removing Li^+^ also weakens the Li^+^−TM^n+^ repulsion, which counteracts with the increasing anion repulsion and eventually results in slight decreases of lattice parameter *c*. Upon further de‐lithiation, the oxygen oxidation reduces the repulsion between the O layers and leads to a faster lattice shrinkage along the *c*‐axis. In the subsequent discharge and charge, lattice parameter *c* changes reversibly with the largest variation of 2.3 %, manifesting that the metastable LLNMO has great structural stability upon de‐/lithiation.

It is noteworthy that asymmetric shoulders were observed over the right of (108) and (110) peaks at the end of the 1^st^ discharge, implying that the metastable R‐3m phase partially converts to a new phase. To further elucidate its structural changes, ex situ XRD patterns (Cu Kα, λ=1.54 Å) were collected on LLNMO electrodes at various states of charge and discharge, wherein a small current density (5 mA/g, ~0.02 C) was used to assure complete phase transformations (Figure S21).

Considering first the (006) peak (Figure 6e, S21c and S22). Upon the initial charge (OCV→1 C–4.0 V and 1 C–4.5 V), it barely changes during the Ni oxidation process. Over the oxygen oxidation plateau (1 C–4.5 V→1 C–4.6 V), it shifts from Q=1.320 Å^−1^ to 1.327 Å^−1^ due to the reduced repulsion between the O layers. So far, the (006) peak is still symmetrical, and no new peaks appear, indicating a single phase deintercalation mechanism. As mentioned before, of particular interest are two reversible voltage plateaus and two sharp dQ/dV peaks in the high potential region (4.6–4.8 V), which have been speculated to undergo biphasic processes. Indeed, over the first plateau (1 C–4.6 V→1 C–4.7 V), the (006) peak shifts slightly to Q=1.328 Å^−1^, while a shoulder at a higher angle (Q=1.340 Å^−1^) can be deconvoluted from the asymmetrical peak profile, manifesting the formation of a new phase with a smaller interlayer spacing. Upon further charging (1 C–4.7 V→1 C–4.8 V), both the (006) peak and the newly formed peak are stable. Those biphasic processes are identical to the formation of O2‐type structures seen in the O2‐type LiCoO_2_.[^47^, ^48^, ^49^]

On discharge (1 D–4.65 V→1 D–4.55 V and 1 D–3.8 V), the newly formed peak disappears, and the symmetrical (006) peak restores at Q=1.326 Å^−1^, which still has a smaller interlayer spacing than that in the OCV state due to the remain of the oxidized O species (Figure S23). During the oxygen reduction (1 D–3.8 V→1 D–3.0 V), it shifts leftward to Q=1.322 Å^−1^. In the final discharge to 1 D–2.0 V, the (006) peak is stable, yet the shoulder emerging at a lower angle (Q=1.317 Å^−1^) indicates the formation of another new phase. Meanwhile, as shown in Figure 6f, a new peak (Q=3.069 Å^−1^) shows up at the left of the (108) peak (Q=3.090 Å^−1^), which can be indexed to (102) peak of an O2‐phase.^[50]^ In the 2^nd^ charge, the new lithiated phase disappears at 2 C–3.0 V and the LLNMO undergoes solid solution processes until over the high potential plateaus (2 C–4.7 V and 2 C–4.8 V), which again witness biphasic processes (Figure S24).

**Figure 7:**
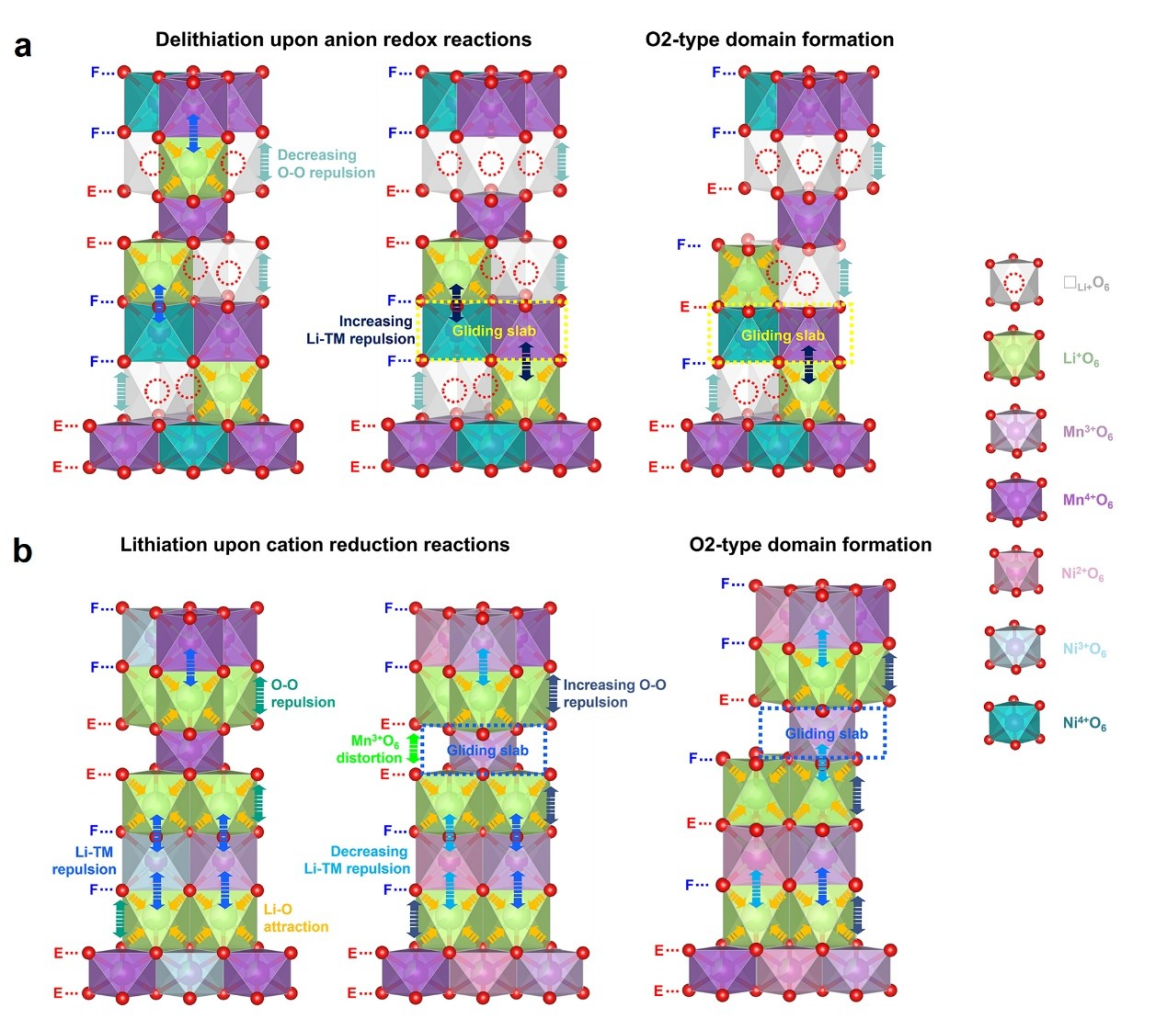
Evolutions of interlayer interactions in LLNMO upon de‐/lithiation. Schematic illustrations of the interlayer interactions upon (a) oxygen oxidation and the formation of the de‐lithiated O2‐phase and (b) cation reduction and the formation of the lithiated O2‐phase. E and F denotes edge‐sharing and face‐sharing sites, respectively.

Overall, in the metastable O6‐type LLNMO, three interlayer interactions (i. e., the Li^+^−O^2−^ attraction, the O^2−^−O^2−^ repulsion, and the Li^+^−TM^n+^ repulsion) determine the interlayer spacings and govern the structural evolutions (Figure S25a). In the initial de‐lithiation (cation oxidation), the O^2−^−O^2−^ repulsion increases due to the diminishing screening effect of Li^+^, which is counteracted by the decreasing Li^+^−TM^n+^ repulsion, thereby resulting in stable interlayer spacing (Figure S25b). Upon further de‐lithiation, the oxygen oxidation reduces the repulsion between the O layers and narrows the interlayer distance between the TMO_2_ slabs. Consequently, the distances between the face‐sharing Li^+^−TM^n+^ ions decrease, leading to a stronger cationic repulsion. To even out the repulsion, the TMO_2_ slabs exclusively face‐sharing with adjacent LiO_2_ slabs (FF‐type) tend to glide along the basal plane (*ab*‐plane), resulting in the partial formation of a de‐lithiated O2‐phase with a smaller interlayer spacing (Figure 7a, S26). Upon lithiation, such a de‐lithiated O2‐phase converts back to the metastable R‐3m phase (O6‐type structure), in which the interlayer interactions further undergo reversible changes via a single‐phase intercalation mechanism until the Mn^4+^‐to‐Mn^3+^ reduction.

Over the Mn reduction in the low potential region (D‐3.0 V→D–2.0 V), the MnO_6_ octahedra are prone to expand in volume because of the weaker covalency of the Mn^3+^−O^2−^ bond than that of the Mn^4+^−O^2−^ bond. From this perspective, the distance between the TMO_2_ slabs narrows and the charge density on O^2−^ increases, both of which induce stronger electrostatic repulsion between the O layers. To relax the increasing repulsion, the TMO_2_ slabs can glide to increase the interlayer spacing via forming more face‐sharing Li^+^−Mn^3+^ sites, producing a lithiated O2‐phase with a larger interlayer spacing (Figure 7b, S27). Such a phase transformation in the deeply lithiated state is also reversible, as evidenced by XRD results. In addition, although it undergoes partial biphasic processes in both the deeply de‐lithiated and lithiated states, the face‐sharing geometry is maintained, meaning that the interlayer TM migration can be suppressed during the entire process.

## Conclusions

In summary, we report a new Co‐free layered oxde cathode material, Li_0.693_[Li_0.153_Ni_0.190_Mn_0.657_]O_2_ (LLNMO), which was prepared through Na^+^/Li^+^ ion exchange and subsequent thermal treatment at moderate temperatures. This material demonstrates exceptional electrochemical properties by tapping into both cationic and anionic redox activities. The oxygen redox activities were meticulously examined using bulk‐sensitive HR‐RIXS, which captured the reversible formation and reduction of molecular O_2_. Detailed structural analyses revealed that it possesses a metastable O6‐type structure characterized by R‐3m symmetry. The face‐sharing local geometry results in strong Li^+^−TM^n+^ repulsion, thereby suppressing interlayer TM migration due to the electrostatic repulsion between face‐sharing cations. Furthermore, the additional interlayer repulsion enhances the stability and flexibility of the metastable LLNMO, allowing it to better accommodate strain accumulation during de‐/lithiation processes. This potentially prevents strain‐induced structural degradations, the loss of trapped O_2_, and the exacerbation of surface‐driven degradation through the formation of intragranular cracks. Given the importance of stabilizing high‐voltage cation and anion redox reactions for the practical application of high‐energy cathode materials, we believe that finely engineering the local structure in a metastable phase to mitigate severe lattice expansion and TM migration is a promising strategy for the design of next‐generation ultra‐high‐energy LIBs.

## Supporting Information

Experimental section, Figure S1–S27, Table S1–S6, and supplementary references.

## Conflict of Interests

The authors declare no conflict of interest.

1

## Supporting information

As a service to our authors and readers, this journal provides supporting information supplied by the authors. Such materials are peer reviewed and may be re‐organized for online delivery, but are not copy‐edited or typeset. Technical support issues arising from supporting information (other than missing files) should be addressed to the authors.

Supporting Information

## Data Availability

The data that support the findings of this study are available from the corresponding author upon reasonable request.
