# Supplementary material for: A Metastable Oxygen Redox Cathode for Lithium‐Ion Batteries
Source: Angew Chem Int Ed Engl. 2025 Feb 11;64(16):e202422789. doi: 10.1002/anie.202422789 (PMC12001160; doi:10.1002/anie.202422789)
Supplement: Supplementary file 1 — Supporting Information [file ANIE-64-e202422789-s001.pdf]

Angewandte Chemie
Eine Zeitschrift der Gesellschaft Deutscher Chemiker

Supporting Information

A Metastable Oxygen Redox Cathode for Lithium-Ion Batteries

Y. Wang, C. Li, Y. Li, R. de Benito, J. Williams, J. M. Stratford, Z. Li, C. Zeng, N. Qin, H. Wang, Y. Cao, D. Gardner, W. Lima da Silva, S. Tippireddy, Q. Gan, F. Zhang, W. Luo, J. W. Makepeace, K.-J. Zhou, K. Zhang, F. Zhang, P. K. Allan*, Z. Lu**

A Metastable Oxygen Redox Cathode for Lithium-Ion Batteries

Yanfang Wang,[a,b,c] Cheng Li,[a] Yingzhi Li,[a] Raquel de Benito,[b] Jacob Williams,[b] Joshua M. Stratford,[b] Zhiqiang Li,[a] Chun Zeng,[a] Ning Qin,[a] Hongzhi Wang,[a] Yulin Cao,[a] Dominic Gardner,[b,d] Wilgner Lima da Silva,[b,d] Sahil Tippireddy,[e] Qingmeng Gan,[a] Fangchang Zhang,[a] Wen Luo,[a] Joshua W. Makepeace,[b] Ke-Jin Zhou,[e] Kaili Zhang,[f] Fucai Zhang,*[c] Phoebe K. Allan,*[b,d] and Zhouguang Lu*[a]

[a] Dr. Y. F Wang, Dr. C. Li, Dr. Y. Z. Li, Dr. Z. Q. Li, C. Zeng, Dr. N. Qin, H. Z. Wang, Y. L. Cao, Dr. Q. M. Gan, F. C. Zhang, Dr. W. Luo, Prof. Z. G. Lu
Department of Materials Science and Engineering
Southern University of Science and Technology
Shenzhen, 518055, China
E-mail: zglu@sustech.edu.cn

[b] Dr. Y. F. Wang, R. Benito, J. Williams, Dr. J. M. Stratford, D. Gardner, Dr. W. L. Silva, Prof. J. W. Makepeace, Prof. P. K. Allan
School of Chemistry
University of Birmingham
Edgbaston, Birmingham, B15 2TT, UK
E-mail: p.allan@bham.ac.uk

[c] Dr. Y. F. Wang, Prof. F. C. Zhang
Department of Electronic and Electrical Engineering
Southern University of Science and Technology
Shenzhen, 518055, China
E-mail: zhangfc@sustech.edu.cn

[d] D. Gardner, Dr. W. L. Silva, Prof. P. K. Allan
The Faraday Institution
Harwell Campus, Didcot, UK

[e] Dr. S. Tippireddy, Dr. K.-J. Zhou
Diamond Light Source
Harwell Campus, Didcot, UK

[f] Prof. K. L. Zhang
Department of Mechanical Engineering
City University of Hong Kong
Kowloon, Hong Kong, China

This file includes Experimental section, Figure S1-S27, Table S1-S6, and Supplementary References.

Experimental section

Materials synthesis. To prepare the P2-type precursor, bimetallic carbonate ($NiMn_3(CO_3)_4$, Hai-An Zhi Chuan Co. Ltd.) was mixed with lithium carbonate (Li_2CO_3, Aladdin, ≥ 99.99 %) and sodium carbonate (Na_2CO_3, Aladdin, ≥ 99.5 %) in a molar ratio of 2: 1.03: 4.12. Then, the mixture was ground by hand and annealed in a muffle furnace. The programmed heating process includes a water removal step at 450 °C for 5 h (5 °C/ min), an annealing period at 800 °C for 12 h (2°C/ min) and a cooling period to 100 °C (2 °C/ min) in air. To obtain the O2+T2 mixture, the P2-type precursor was mixed with $LiNO_3$ (Aladdin, ≥ 99.9 %) and LiCl (Aladdin, ≥ 99.99 %) in a weight ratio of 1: 8.2 :1.8 and annealed in a muffle furnace at 280 °C for 4 h in air. The as-obtained product was washed thoroughly with distilled water and dried at 80 °C overnight. Finally, to get the metastable LLNMO, the dried O2+T2 mixture was annealed at 500 °C for 12 h in air.

General characterizations. The morphology of the as-synthesized materials was characterized by using scanning electron microscopy (SEM) (TESCAM MIRA 3), transmission electron microscope (TEM) (Talos F200X) and spherical aberration corrected transmission electron microscope (ACTEM) (Titan Themis G2). Raman spectra were collected on a Renishaw inVia Raman spectrometer. X-ray photoelectron spectroscopy (XPS) data were collected on a PHI5300 X-ray photoelectron spectrometer using Mg Kα radiation (250 W, 14 kV). Powder X-ray diffraction (XRD) was performed on a Rigaku X-ray diffractometer (Rigaku, Japan) with a Cu Kα radiation (λ=1.54 Å, 9 kW). Thermal gravimetric analysis-mass spectrometry (TGA-MS) test was performed on STA449F3-QMS403D (Netzsch, Germany). Magnetic property was studied with a SQUID magnetometer (MPMS-3). The TOF neutron diffraction pattern was collected on a multi-purpose neutron reflectometer (BL02) at China Spallation Neutron Source (CSNS). Rietveld refinement was performed by using GSAS-EXPGUI and GSAS-II.[1,2] Synchrotron XRD patterns of the P2-precursor and the O2+T2 mixture were collected on BL14B (E=18 KeV, λ=0.6887 Å) at Shanghai Synchrotron Radiation Facility (SSRF). *Operando* synchrotron XRD data were collected on I15-1 (E=76.69 KeV, λ=0.16167 Å) at Diamond Light Source (DLS).

Cell assembly and electrochemistry. The as-prepared materials, carbon black and polyvinylidene fluoride (PVDF) in the weight ratio of 8: 1: 1 were mixed homogeneously in N-methyl pyrrolidone (NMP) and coated on an aluminum foil (12 μm in thickness) before cutting it into pieces (1.2 cm in diameter). CR2016-type coin cells were assembled in an argon-filled glovebox by using the as-prepared electrodes (cathode, ~50 μm in thickness), lithium metal plates (anode, 200 μm in thickness) and Celgard membranes (separator, 25 μm in thickness). The areal mass loading of active material is around 2.0 mg/ cm^2. The fluorinated electrolyte is a solution of 1 M $LiPF_6$ (Battery grade, Innochem) in a mixture of fluoroethylene carbonate (FEC, Innochem), 3,3,3-fluoroethylmethyl carbonate (FEMC, Macklin), and 1,1,2,2-tetrafluoroethyl-2′,2′,2′-trifluoroethyl ether (HFE, Innochem) (2:6:2 in volume) with the additive of 2 wt% lithium difluoro(oxalato)borate (LiDFOB, Macklin). The ordinary electrolyte is a solution of 1 M $LiPF_6$ in ethylene carbonate (EC)/ dimethyl carbonate (DMC) binary solvent (1:1 in volume). The amount of electrolyte injected for each coin-cell was around 40 μL in volume. Galvanostatic charge-discharge (GCD) and electrochemical impedance spectroscopy (EIS) tests were performed on a BioLogic electrochemical workstation. Long time cycling tests at 50 and 100 mA/g were performed on the Neware battery cycler (CT-4008-5V-10mA-164, Shenzhen, China), following 5 cycles of GCD tests at 10 mA/g for activation. All tests were performed at 25 °C.

***Ex-situ* XRD, XPS, XAS and EPR.** To prepare electrodes at different electrochemical states, the above-mentioned coin cells were dis-/charged to different voltages and maintained at corresponding voltages for

30 min, before being disassembled in the argon protected glove box, washed with dimethyl carbonate (DMC) and naturally dried in argon flow. *Ex-situ* XRD tests were performed on a Rigaku X-ray diffractometer (Rigaku, Japan) with a Cu Kα radiation (λ=1.54 Å, 9 kW). For XPS tests, the as-obtained electrodes were vacuum transferred to chambers for XPS tests. The raw data were processed (normalization and fitting) by using XPSPEAK41 software. The peak position correction was applied based on the C 1s peak (284.8 eV). For EPR tests, the cycled cathode materials were transferred into paramagnetic tubes and tested at room temperature with an EMX-plus 10/12 spectrometer of the X-band Bruker. For XAS tests, the Mn K-edge and Ni K-edge XANES spectra were collected at beamline 11B of Shanghai Synchrotron Radiation Facility (SSRF).

HR-RIXS and SXAS. High resolution resonant inelastic X-ray scattering (HR-RIXS) for the O K-edge were collected at the I21 beamline at Diamond Light Source.[3] Samples were transferred to the spectrometer using a vacuum-transfer suitcase to avoid air exposure and were pumped down to ultra-high vacuum (UHV) and left to fully degas overnight. RIXS maps were measured in 0.2 eV energy increments from 526 eV to 536 eV at five different sample locations summed together. RIXS line scans were recorded at the resonance energy for molecular O_2 (530.5 eV) at 15 different sample locations and averaged together. All data were obtained in partial fluorescence mode for bulk sensitivity. All measurements were performed at 20 K to minimize any possible beam damage.

***In situ* VT-XRD and *operando* synchrotron XRD.** *In-situ* varied temperature (VT)-XRD test for O2+T2 mixture (powder) were performed on a Rigaku X-ray diffractometer (Rigaku, Japan) with a Cu Kα radiation (λ=1.54 Å, 9 kW) and a one-dimensional detector was used to increase the intensity of diffractive signals. Specifically, the O2+T2 mixture was placed on the Al_2O_3 holder and transferred into the high-temperature chamber. The sample was heated from room temperature (R.T., 25 °C) to 800 °C (5 °C/ min) with a holdup time of 30 minutes for every 25 °C to collect XRD patterns. *Operando* synchrotron XRD data were collected on I15-1 (E=76.69 KeV, λ=0.16167 Å,) at Diamond Light Source (DLS). A customized Diamond Radial In Situ X-ray (DRIX) cell was used,[4] which was assembled in an argon protected glovebox.

***Ex-situ* and *operando* Raman spectroscopy.** Electrodes for *ex-situ* Raman tests were prepared in similar ways as mentioned above. A customized *in-situ* cell with a quartz window was used for *operando* test.[5] To avoid possible damage caused by the laser, the power of the laser and the exposure time were chosen deliberately upon multiple trials. For the laser (532 nm) used for *operando* test, an exposure time of 10 s and the 5 % of the maximum power were used. It is noteworthy that even the same sample presents different tolerance towards different lasers, meaning that the choice of laser for *operando* Raman tests should be considered case by case. The corresponding electrochemical performance of the *in-situ* cell was recorded by using an electrochemical workstation (CHI660E, Shanghai, China).

	**7 Li [He]**		**23 Na [He]**		**55 Mn [He]**		**60 Ni [He]**	
	Conc. [ppb]	Conc. RSD	Conc. [ppb]	Conc. RSD	Conc. [ppb]	Conc. RSD	Conc. [ppb]	Conc. RSD
**P2-precursor**	59.8561(9)	4.8649(2)	610.9816(5)	3.1460(6)	1291.0716(5)	2.5362(7)	405.1408(8)	2.5272(8)
**LLNMO**	278.38	1.0964(1	<0.000	N/A	1697.29	0.8950(5	537.539	0.8659(2

24(9)	)	55(5)	)	2(7)	)

Table S1. ICP-MS results

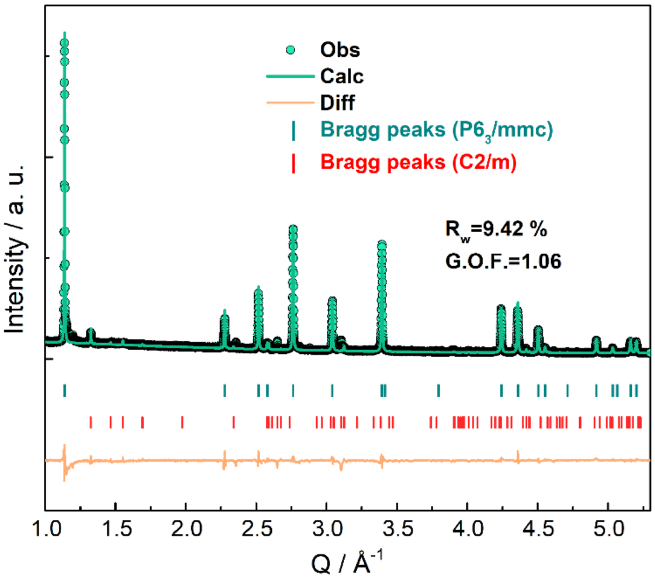

Figure S1. Rietveld refinement of synchrotron XRD patterns (λ=0.6887 Å) of the P2-type precursor. The precursor is mainly composed of a P2-phase (95.9 wt%) with a small impurity of Li_2MnO_3 (4.1 wt%).

Table S2. Rietveld refinement results of the P2-type precursor

**Phase 1: $Na_{0.745}[Li_{0.153}Ni_{0.194}Mn_{0.626}]O_2$**			Space group: P 6_3/mmc		wt=95.9%
Lattice parameters: a=2.8831(6) Å, b=2.8831(6) Å, c=11.0418(1) Å; α=90°, β=90°, γ=120°; $V_{unit\ cell}$=79.49(1) Å^3					
Atom	Fractional coordinates x	y	z	Multiplicity	Occupancy
Na1	0	0	1/4	2	0.237(1)
Na2	1/3	2/3	1/4	2	0.475(1)
Li1	0	0	0	2	0.191(1)
Li2	0	0	1/4	2	0.039(1)
Mn1	0	0	0	2	0.628(1)
Ni1	0	0	0	2	0.181(1)
O1	2/3	1/3	0.0923(6)	4	1
**Phase 2: Li_2MnO_3**			Space group: C 2/m		wt=4.1%
Lattice parameters: a=4.9199(8) Å, b=8.5775(6) Å, c=5.0801(5) Å; α=90°, β=111.05(5)°, γ=90°; $V_{unit\ cell}$=200.07(6) Å^3					
Atom	Fractional coordinates x	y	z	Multiplicity	Occupancy
Li1	0	1/2	0	2	1
Li2	0	0	1/2	2	1
Li3	0	0.6606(1)	1/2	4	1
Mn1	0	0.1670(8)	0	4	1
O1	0.2189(1)	0	0.2273(1)	4	1
O2	0.2541(1)	0.3211(9)	0.2233(1)	8	1
Refinement results: R_w=9.42%, G.O.F.=1.06, Reduced χ^2=1.12					

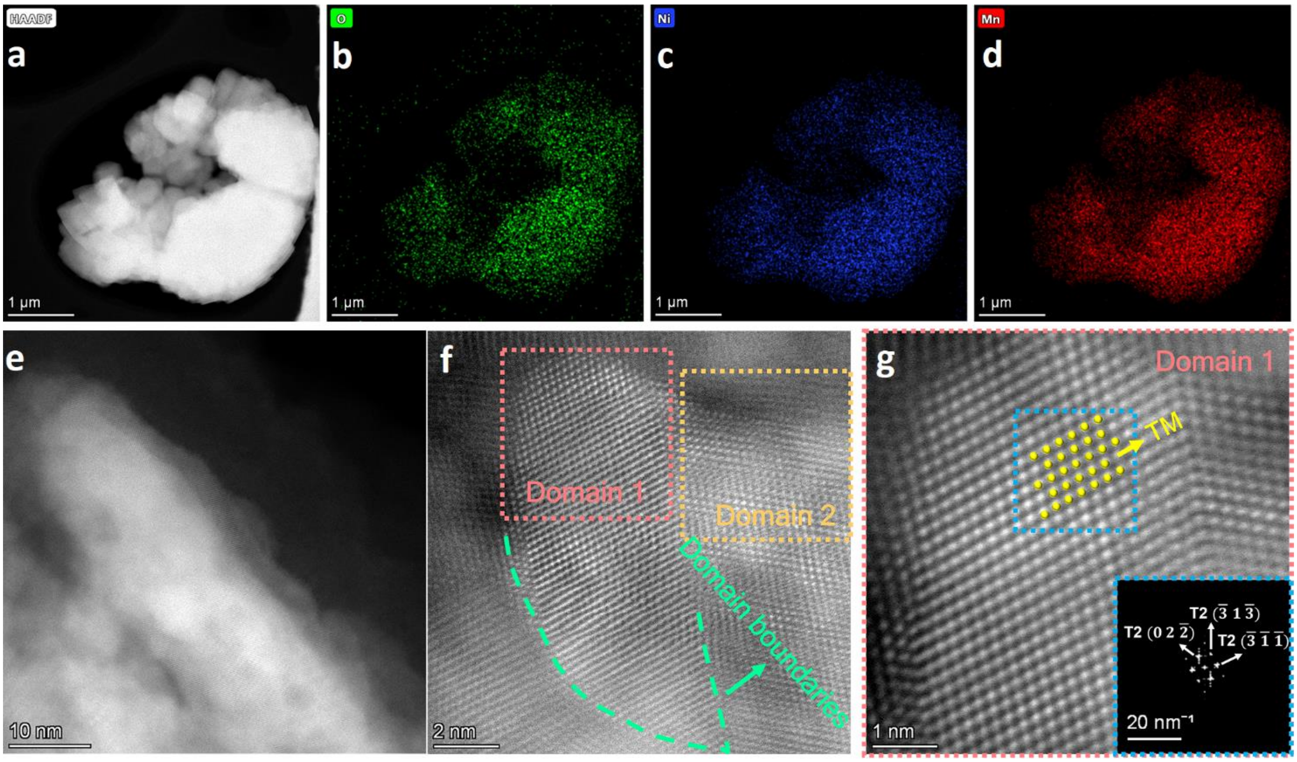

Figure S2. Morphology of the O2+T2 mixture. (a) HAADF-STEM image and (b-d) energy dispersive X-ray (EDX) mappings. All elements disperse uniformly. (e) Cs-corrected HAADF-STEM image. Its crystallinity seems to be poor due to the lack of long fringes. (f, g) Enlarged images showing nanodomains belonging to different phases or the same phase with different orientations, wherein the green dashed lines represent domain boundaries. The inset of (g) displays fast Fourier transform (FFT) patterns of the selected area, which could be indexed to a T2-phase. Yellow balls represent TM ions.

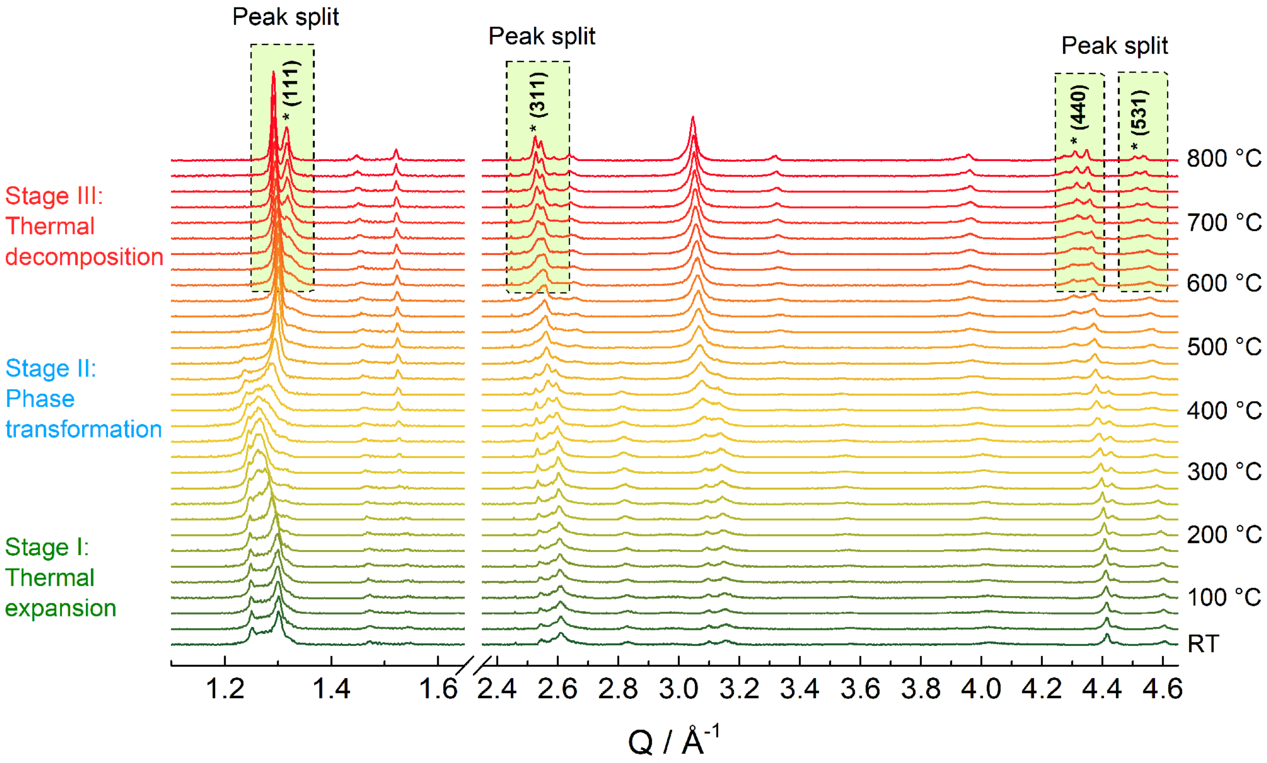

Figure S3. *In situ* VT-XRD patterns (λ=1.54 Å) for the O2+T2 mixture. Peaks labeled by asterisks can be indexed to a spinel phase (Fd-3m symmetry).

At room temperature (R.T.), XRD peaks at around Q = 1.25 Å^{-1} and 1.30 Å^{-1} correspond to (002) peaks of T2- and O2-phases, respectively. In the region of R.T. to 300 °C, the O2-(002) peak shifts to lower Q dure to thermal expansion. Further heating up to 400 °C induces phase transformation and produces a material with a different symmetry. Such an intermediate phase is metastable and decomposes into two phases at higher temperatures (> 600 °C), as indicated by the split of the diffraction peaks.

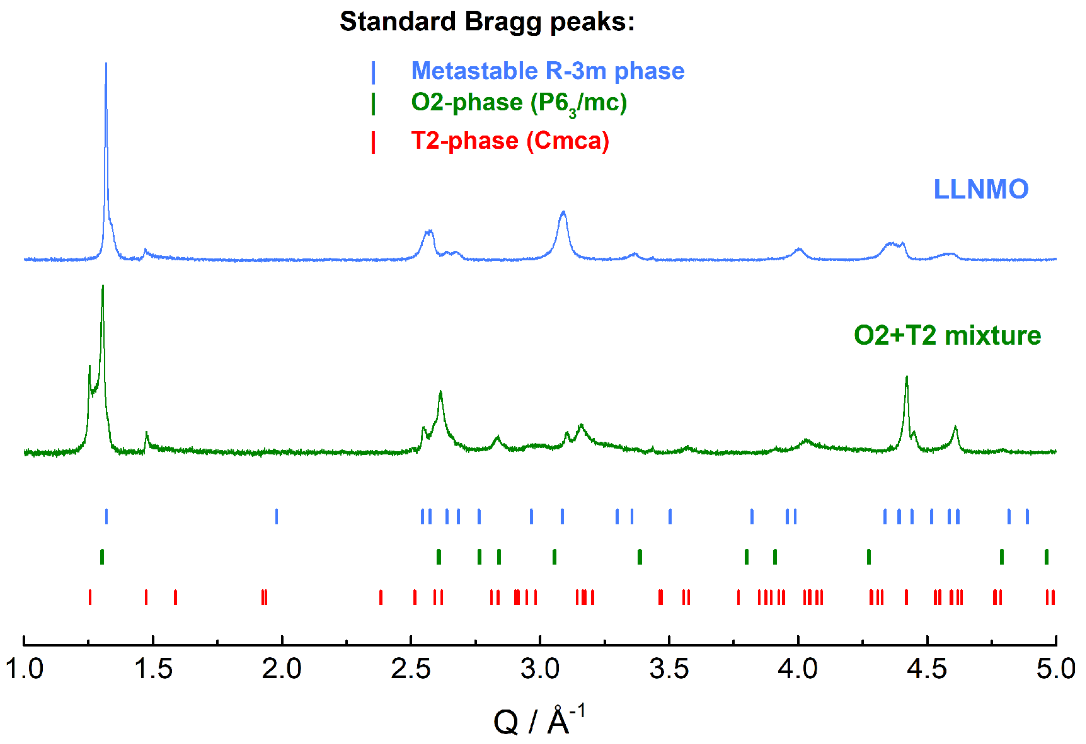

Figure S4. Synchrotron XRD patterns (λ=0.6887 Å) of the O2+T2 mixture and the metastable LLNMO. For both samples, some unindexed peaks belong to the small impurity of Li_2MnO_3, which already exists in the P2-precursor.

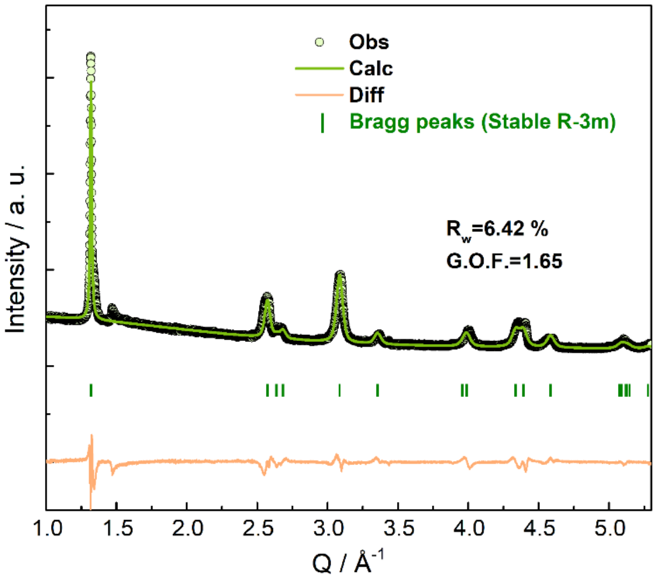

Figure S5. Rietveld refinement of synchrotron XRD patterns (λ=0.6887 Å) of the LLNMO with an O3-type model (R-3m symmetry).

Table S3. Rietveld refinement results of the LLNMO with an O3-type model

**Phase: $Li_{0.693}[Li_{0.153}Ni_{0.190}Mn_{0.657}]O_2$**			Space group: R -3m		wt=100%
Lattice parameters: a=2.8624(1) Å, b=2.8624(1) Å, c=14.2881(7) Å; α=90°, β=90°, γ=120°; $V_{unit\ cell}$=101.38(4) $Å^3$					
Atom	Fractional coordinates x	y	z	Multiplicity	Occupancy
Li1	0	0	0	3	0.693
Li2	0	0	1/2	3	0.153
Mn1	0	0	1/2	3	0.657
Ni1	0	0	1/2	3	0.19
O1	0	0	0.2402(6)	6	1
Refinement results: R_w=6.42%, G.O.F.=1.65, Reduced χ^2=2.72					

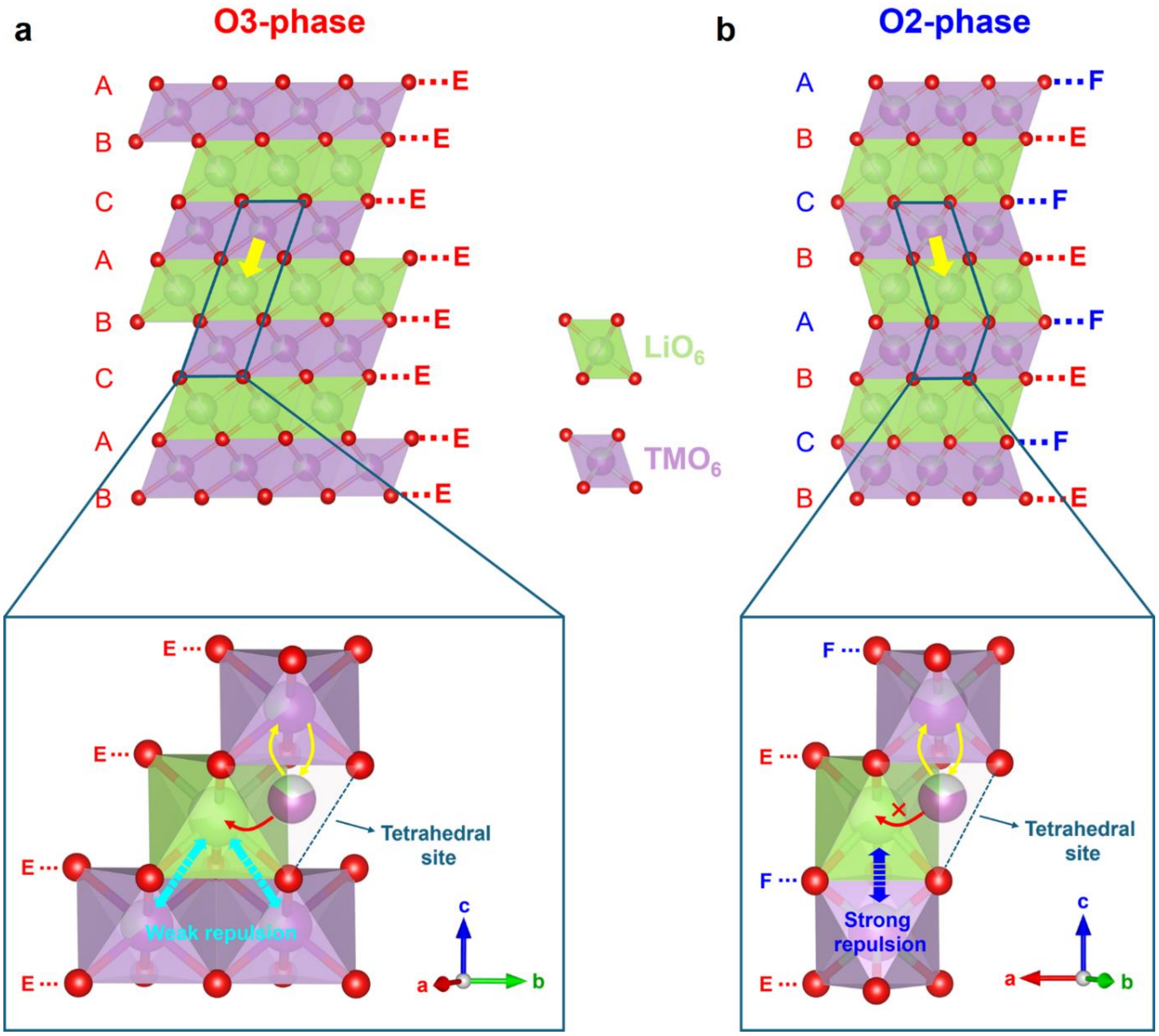

Figure S6. Comparison of crystal structures and TM migration paths. Schematic illustrations of (a) O3-type and (b) O2-type structures. The figures below show the TM migration paths on a magnified scale. E and F denotes edge-sharing and face-sharing sites, respectively.

In the O3-type structure, along the *c*-axis, the LiO_2 slabs only share edges with adjacent TMO_2 slabs. In the O2-type structure, along the *c*-axis, the LiO_2 slabs share edges with adjacent TMO_2 slabs on one side and share faces with others on the other side. In addition, TMO_2 slabs in the O3-type structure display the same orientation, whereas they display alternating orientations in the O2-type structure (i.e., the two neighboring TMO_2 slabs have different orientations).

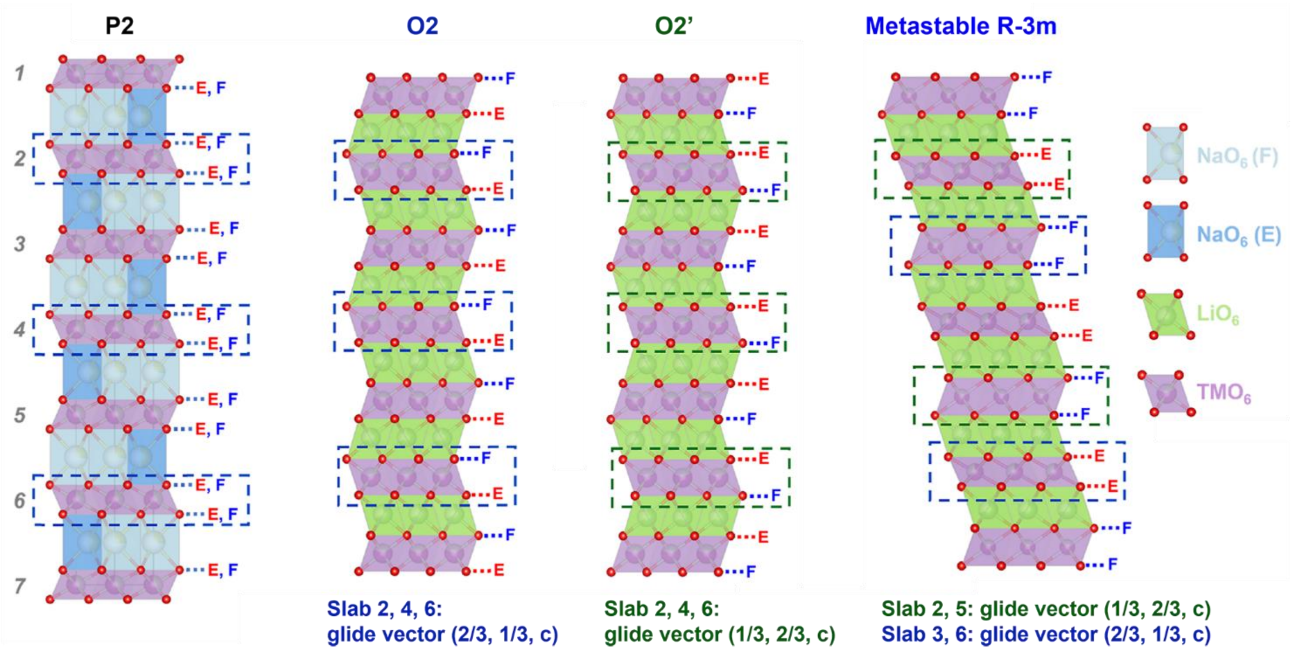

Figure S7. Schematic illustration of forming the metastable R-3m phase. E and F denote edge-sharing and face-sharing planes between prismatic NaO_6/octahedral LiO_6 and octahedral TMO_6, respectively.

Via Na^+/Li^+ ion exchange, to form the O2-type structure from the P2-type precursor, TMO_2 slabs with odd numbers (i.e., 1, 3, 5) stay at their original positions, whereas those with even numbers (i.e., 2, 4, 6) glide towards the (2/3, 1/3, *c*) direction, while Na^+ ions at prismatic sites are replaced by Li^+ ions at octahedral sites. It is noteworthy that TMO_2 slabs with even numbers (i.e., 2, 4, 6) possibly glide toward the (2/3, 1/3, *c*) direction, producing the O2'-type structure. Both the O2- and the O2'-type structures are superimposed with each other via rotating 180° with the axis of symmetry.[6] In the ideal O2- and O2'-type structures, a LiO_6 octahedron shares a face with a TMO_6 octahedron on the one side and shares three edges with three TMO_6 octahedra on the other side, and vice versa.

As for the metastable R-3m phase, it is arguably an intergrowth between the O2- and O2'-phases. Specifically, for every three adjacent TMO_2 slabs, every the second TMO_2 slab glides toward the (1/3, 2/3, *c*) direction and every the third TMO_2 slab glides toward the (2/3, 1/3, *c*) direction (e.g., TMO_2 slabs numbered as 1 and 4 stay at their original positions; TMO_2 slabs numbered as 2 and 5 glide towards the (1/3, 2/3, *c*) direction; TMO_2 slabs numbered as 3 and 6 glide towards the (2/3, 1/3, *c*) direction). As revealed by *in-situ* VT-XRD and TGA-MS results, the phase transformation between O2-/O2'-type and the metastable R-3m structures can happen via the gliding of TMO_2 slabs without breaking the TM-O bonds or releasing gaseous O_2.

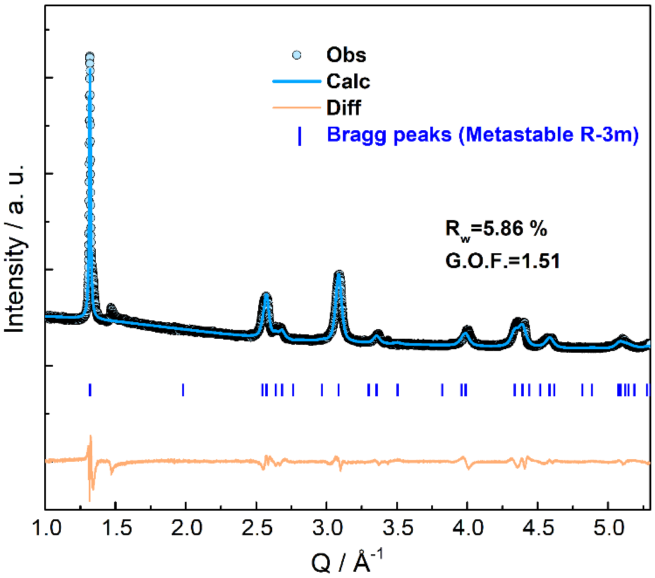

Figure S8. Rietveld refinement of synchrotron XRD patterns (λ=0.6887 Å) of the LLNMO with a metastable rhombohedral model (R-3m symmetry).

Table S4. Rietveld refinement results of the LLNMO with a metastable rhombohedral model

**Phase: $Li_{0.693}[Li_{0.153}Ni_{0.190}Mn_{0.657}]O_2$**			Space group: R -3m		wt=100%
Lattice parameters:<br>a=2.8618(7) Å, b=2.8618(7) Å, c=28.5728(9) Å; α=90°, β=90°, γ=120°; $V_{unit\ cell}$=202.66(7) Å^3					
Atom	Fractional coordinates			Multiplicity	Occupancy
	x	y	z		
Li1	0	0	1/12	6	0.693
Li2	0	0	0	3	0.153
Li3	0	0	1/2	3	0.153
Mn1	0	0	0	3	0.657
Mn2	0	0	1/2	3	0.657
Ni1	0	0	0	3	0.19
Ni2	0	0	1/2	3	0.19
O1	0	0	0.2038(1)	6	1
O2	0	0	0.3682(1)	6	1
Refinement results: R_w=5.86%, G.O.F.=1.51, Reduced χ^2=2.27					

Table S5. Rietveld refinement results of the LLNMO with two phases

**Phase 1: $Li_{0.693}[Li_{0.153}Ni_{0.190}Mn_{0.657}]O_2$**			Space group: R -3m		wt=95.4%
Lattice parameters: a=2.8651(5) Å, b=2.8651(5) Å, c=28.5858(7) Å; α=90°, β=90°, γ=120°; $V_{unit\ cell}$=203.22(5) Å^3					
Atom	Fractional coordinates x	y	z	Multiplicity	Occupancy
Li1	0	0	1/12	6	0.693
Li2	0	0	0	3	0.153
Li3	0	0	1/2	3	0.153
Mn1	0	0	0	3	0.657
Mn2	0	0	1/2	3	0.657
Ni1	0	0	0	3	0.19
Ni2	0	0	1/2	3	0.19
O1	0	0	0.2044(5)	6	1
O2	0	0	0.3685(4)	6	1
**Phase 2: Li_2MnO_3**			Space group: C 2/m		wt=4.6%
Lattice parameters: a=5.0160(7) Å, b=8.5146(4) Å, c=4.9462(7) Å; α=90°, β=108.705(1)°, γ=90°; $V_{unit\ cell}$=200.09(7) Å^3					
Atom	Fractional coordinates x	y	z	Multiplicity	Occupancy
Li1	0	1/2	0	2	1
Li2	0	0	1/2	2	1
Li3	0	0.6606(1)	1/2	4	1
Mn1	0	0.1670(8)	0	4	1
O1	0.2189(1)	0	0.2273(1)	4	1
O2	0.2541(1)	0.3211(1)	0.2233(1)	8	1
Refinement results: R_w=3.96%, G.O.F.=1.02, Reduced χ^2=1.03					

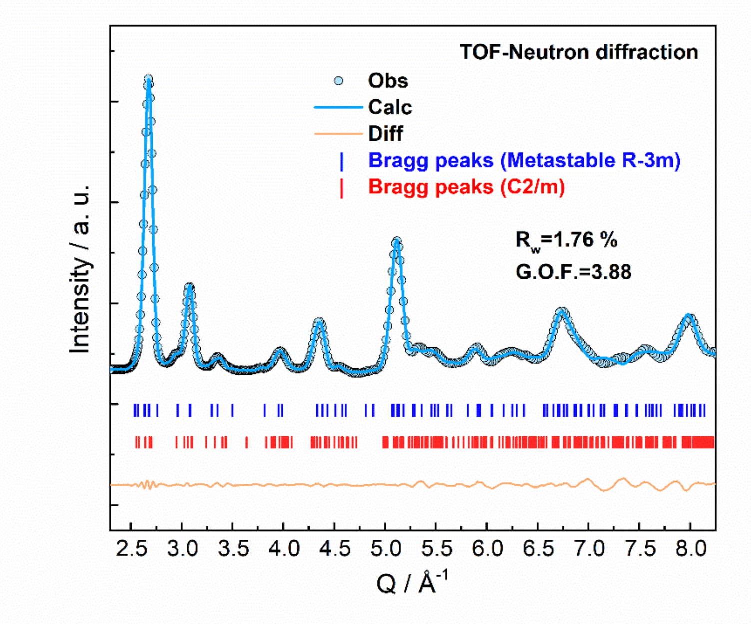

Figure S9. Rietveld refinement of TOF neutron diffraction patterns of the LLNMO with a metastable structure (R-3m symmetry, 95.4 wt%) and the impurity of Li_2MnO_3 (C2/m symmetry, 4.6 wt%).

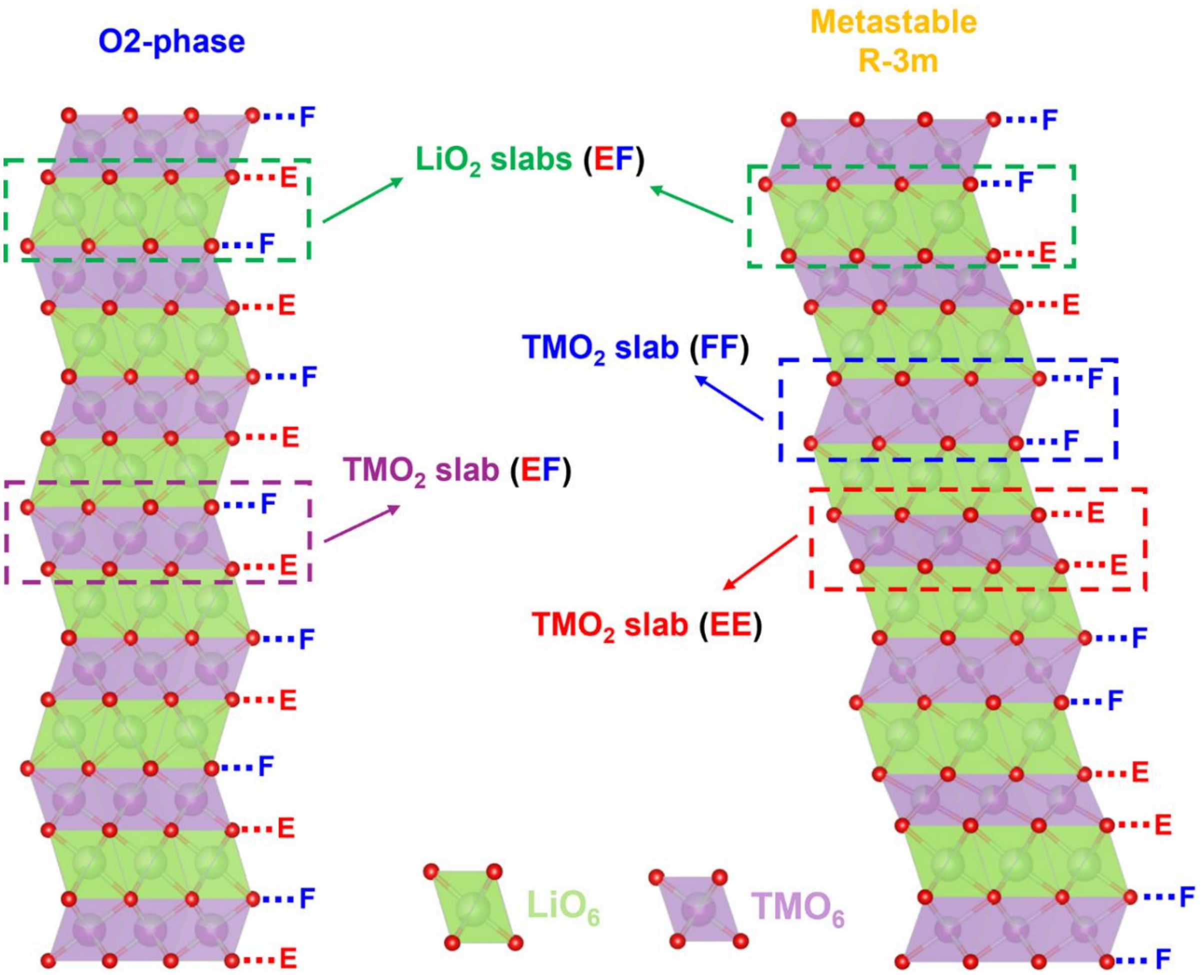

Figure S10. Schematic illustration of local structures in the O2-phase (left) and the metastable R-3m phase (right). E and F denotes edge-sharing and face-sharing sites, respectively.

From the perspective of local geometry, for the LiO_2 slabs in both the O2-type structure and the metastable R-3m structure, they share faces with adjacent TMO_2 slabs on one side and share edges with the others on the other side (denoted as EF-type). However, for the TMO_2 slabs, they have both face-sharing and edge-sharing patterns in the O2-phase (EF-type), whereas they are either exclusively face-sharing (FF-type) or exclusively edge-sharing (EE-type) with adjacent LiO_2 slabs in the metastable R-3m structure.

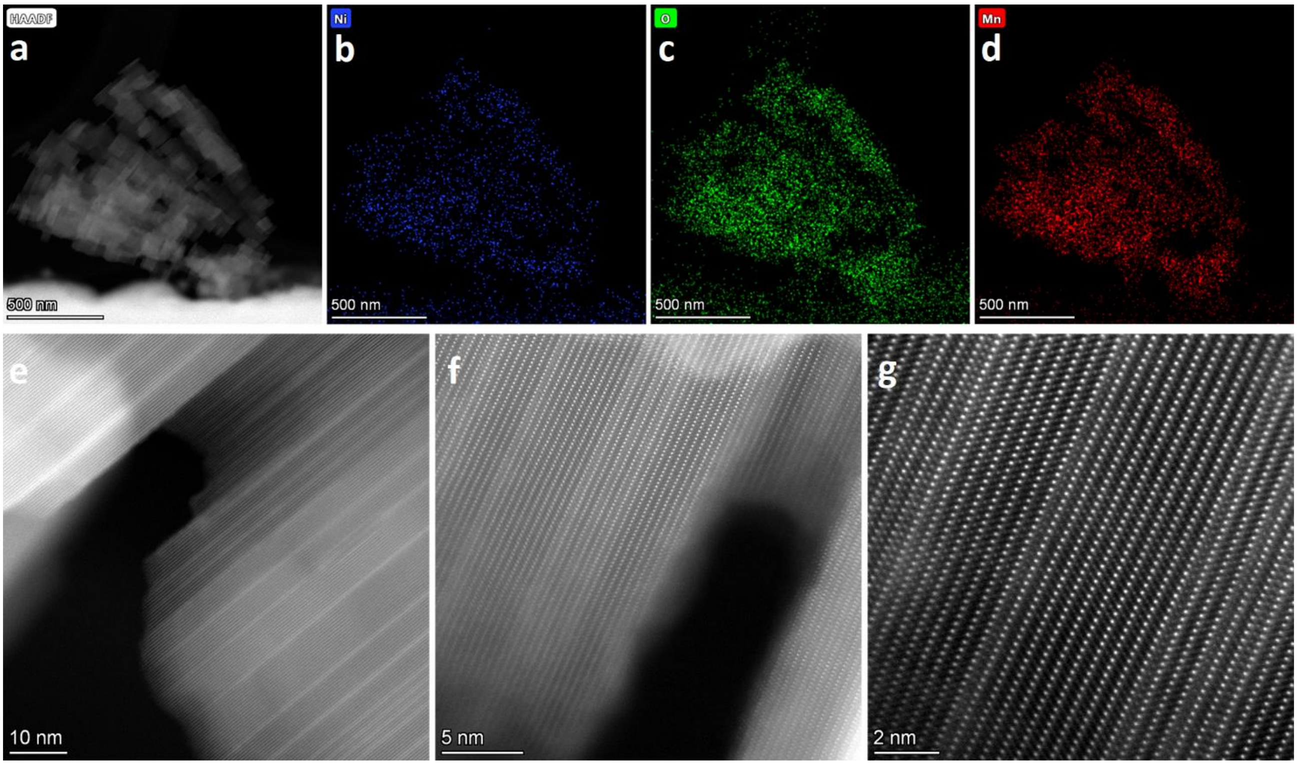

Figure S11. Morphology of the metastable LLNMO. (a) HAADF-STEM image and (b-d) energy dispersive X-ray (EDX) mappings. All elements disperse uniformly. (e-g) Cs-corrected HAADF-STEM image showing the layered structure and nanodomains. Its crystallinity is better than that of the O2+T2 mixture, as revealed by the appearance of long fringes.

Table S6. Cycling performance of cobalt-free O2-/O3-type cathodes and the metastable LLNMO

Phase	Composition	Initial capacity	Capacity retention	Ref.
O2	$Li_x[Li_{0.2}Mn_{0.8}]O_2$	230 mAh/g, 2.0-4.7 V, 10 mA/g	198.0 mAh/g, 86.1 %, 80 cycles	7
	$Li[Li_{0.25}Mn_{0.75}]O_2$	216 mAh/g, 2.0-4.8 V, 250 mA/g	171.1 mAh/g, 79.2 %, 100 cycles	8
	$Li_{0.72}[Li_{0.12}Ni_{0.36}Mn_{0.52}]O_2$	184 mAh/g, 2.0-4.8 V, 10 mA/g	139.8 mAh/g, 76.0 %, 100 cycles	9
	$Li_{2/3}[Ni_{0.25}Mn_{0.75}]O_2$	203 mAh/g, 2.0-4.8 V, 28 mA/g	129.9 mAh/g, 64.0 %, 50 cycles	10
	$Li_{0.83}[Li_{0.2}Ni_{0.2}Mn_{0.6}]O_2$	210 mAh/g, 2.0-4.8 V, 5 mA/g	174.9 mAh/g, 83.3 %, 40 cycles	11
	$Li_{5/6}[Li_{0.2}Ni_{0.2}Mn_{0.6}]O_2$	249 mAh/g, 2.0-4.8 V, 5 mA/g	194.2 mAh/g, 78 %, 20 cycles	12
	$Li_{2/3}[Ni_{0.25}Mn_{0.75}]O_2$	188 mAh/g, 2.0-4.8 V, 28 mA/g	171.1 mAh/g, 91 %, 50 cycles	13
	$Li_x[Li_{0.25}Ni_{0.05}Mn_{0.7}]O_2$	246 mAh/g, 2.0-4.6 V, 20 mA/g	145.1 mAh/g, 59 %, 100 cycles	14
	$Li_x[Li_{0.225}Ni_{0.095}Mn_{0.68}]O_2$	238 mAh/g, 2.0-4.6 V, 20 mA/g	157.1 mAh/g, 66 %, 100 cycles	14
	$Li_x[Li_{0.2}Ni_{0.14}Mn_{0.66}]O_2$	230 mAh/g, 2.0-4.6 V, 20 mA/g	186.3 mAh/g, 81 %, 100 cycles	14
	$Li_x[Li_{0.175}Ni_{0.185}Mn_{0.64}]O_2$	224 mAh/g, 2.0-4.6 V, 20 mA/g	201.6 mAh/g, 90 %, 100 cycles	14
	$Li_x[Li_{0.15}Ni_{0.23}Mn_{0.62}]O_2$	220 mAh/g, 2.0-4.6 V, 20 mA/g	195.8 mAh/g, 89 %, 100 cycles	14
O3	$Li_{2/3}[Ni_{0.25}Mn_{0.75}]O_2$	216 mAh/g, 2.0-4.8 V, 28 mA/g	138.2 mAh/g, 64.0 %, 50 cycles	10
	$Li[Li_{0.2}Ni_{0.2}Mn_{0.6}]O_2$	220 mAh/g, 2.0-4.8 V, 5 mA/g	155.1 mAh/g, 70.5 %, 40 cycles	11
	$Li[Li_{0.198}Ni_{0.188}Mn_{0.614}]O_2$	250 mAh/g, 2.0-4.8 V, 100 mA/g	226.8 mAh/g, 90.7 %, 100 cycles	15
	$Li_{4/7}[\square_{1/7}Mn_{6/7}]O_2$	~175 mAh/g, 2.0-4.8 V, 300 mA/g	~150 mAh/g, ~85.7 %, 100 cycles	16
	$Li_{0.6}[Li_{0.2}Mn_{0.8}]O_2$	~175 mAh/g, 2.0-4.8 V, 300 mA/g	~155 mAh/g, 88.6 %, 100 cycles	17
This work	$Li_{0.693}[Li_{0.153}Ni_{0.190}Mn_{0.657}]O_2$	194.9 mAh/g, 2.0-4.8 V, 100 mA/g	**187.1 mAh/g, 96.0 %**, 100 cycles	
	$Li_{0.693}[Li_{0.153}Ni_{0.190}Mn_{0.657}]O_2$	222.4 mAh/g, 2.0-4.8 V, 50 mA/g	**218.2 mAh/g**, **97.4 %**, 50 cycles	

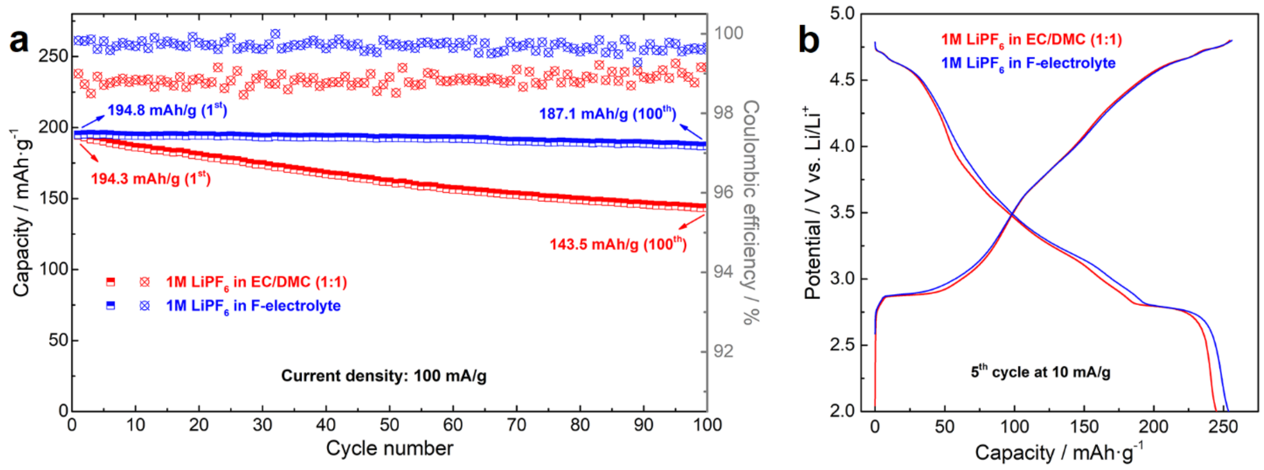

Figure S12. Electrochemistry of the metastable LLNMO in different electrolytes. (a) Cycling performances at the current density of 100 mA/g within the potential window of 2.0-4.8 V vs Li/Li^+ (after 5 cycles at 10 mA/g for activation). (b) Voltage profiles in the 5th cycle at 10 mA/g.

Upon cycling at the current density of 100 mA/g (**Figure S12a**), the initial capacities of the metastable LLNMO electrode in the fluorinated electrolyte (F-electrolyte) and the ordinary electrolyte are almost the same, i.e., 194.8 mAh/g in the former and 194.3 mAh/g in the latter. In addition, the voltage profiles of the metastable LLNMO are identical in those two electrolytes (**Figure S12b**), meaning that testing in the F-electrolyte does not change its intrinsic electrochemistry. However, the coulombic efficiency of the LLNMO electrode is only around 99.0 % in the ordinary electrolyte, which is higher than 99.5 % in the F-electrolyte. The low coulombic efficiency implies the continuous consumption of the electrolyte. Therefore, after 100 cycles in the ordinary electrolyte, only 73.9 % capacity is retained, while the LLNMO electrode shows a 96.0 % capacity retention upon cycling in the F-electrolyte. Considering that no surface modification was employed, the bared surface of the metastable LLNMO cathode can react with the electrolyte severely due to its significant oxygen redox activities, resulting in performance degradation.

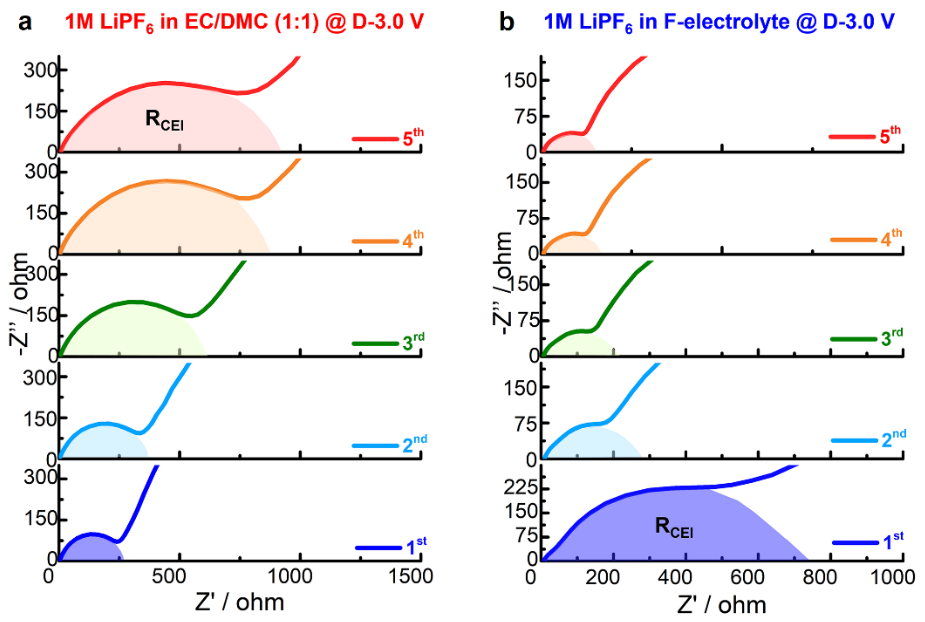

Figure S13. Nyquist plots of the metastable LLNMO in different electrolytes. The tests were performed at 3.0 V in discharge (D-3.0 V) over the initial 5 cycles. Solid lines are fitted from experimental results and colored semicircles depict resistances of the cathode-electrolyte interphase (R_{CEI}).

When tested in an ordinary electrolyte, the resistance of the cathode-electrolyte interphase (R_{CEI}) keeps increasing due to continuous electrolyte consumption, whereas it gradually decreases and stabilizes after 5 cycles in the F-electrolyte.

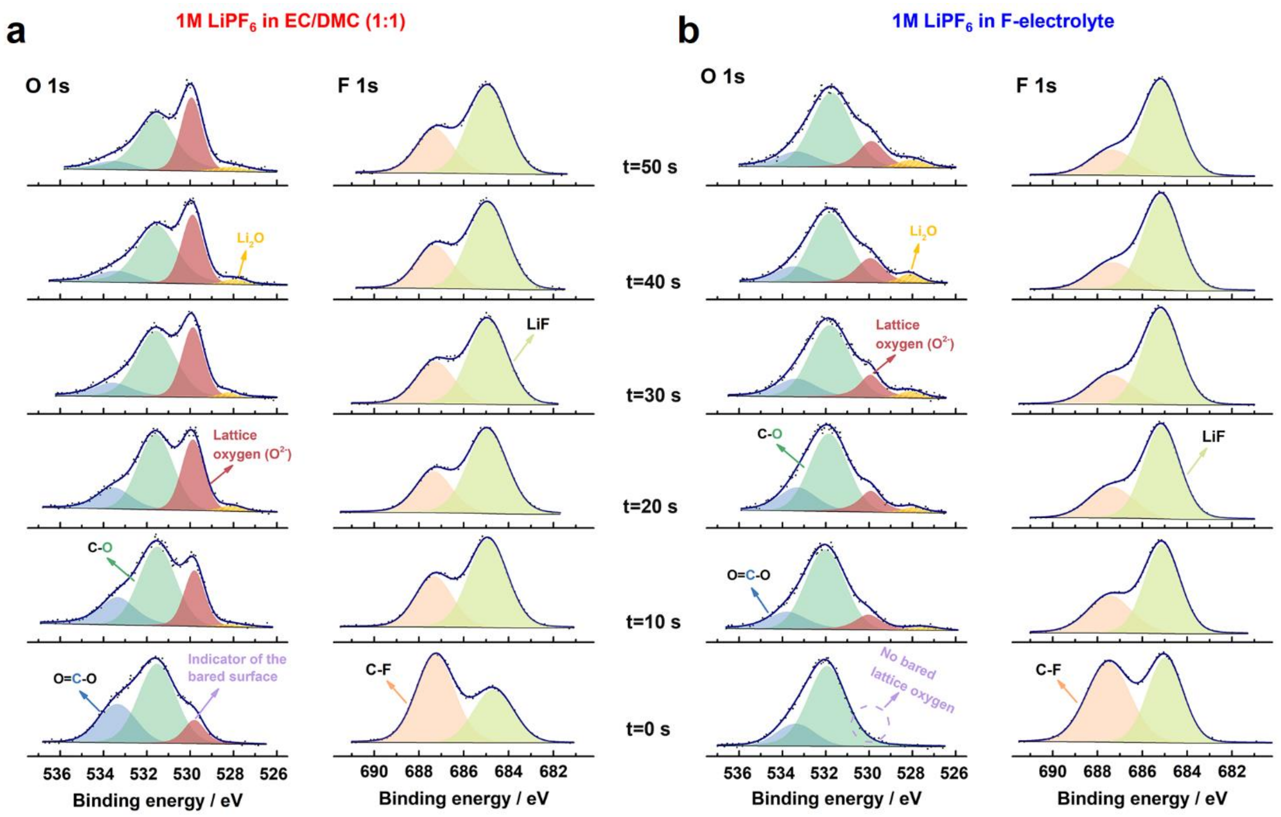

Figure S14. XPS depth profiling of the LLNMO electrodes after 5 cycles in different electrolytes. From bottom to top, the electrodes were etched by Ar^+ beam for 0, 10, 20, 30, 40 and 50 s, respectively. The etching speed is estimated to be 0.2 nm/s. The peak position correction was applied based on the C 1s peak (284.8 eV).

In the pristine O 1s spectrum, the shoulder at 529.8 eV reveals the bared lattice oxygen for the electrode cycled in the ordinary electrolyte, whereas it is not observed in the electrode cycled in the F-electrolyte. Peaks at around 531.5 eV and 533.5 eV are attributed to surface adsorbates and oxidized species, while weak peaks at around 528 eV are attributed to Li_2O.[18] Upon surface etching, both electrodes show increasing amounts of bared lattice oxygen, yet the intensity of the peak at 529.8 eV is much higher for the electrode cycled in the ordinary electrolyte than that of the one cycled in the F-electrolyte. In addition, the much stronger LiF peaks (684.7 eV) compared to the C-F bonds (687.4 eV) demonstrate that the CEI formed in the F-electrolyte is LiF-enriched,[19,20] meaning that they can protect the electrode from being continuously corroded by the electrolyte especially when oxygen redox activities take place.

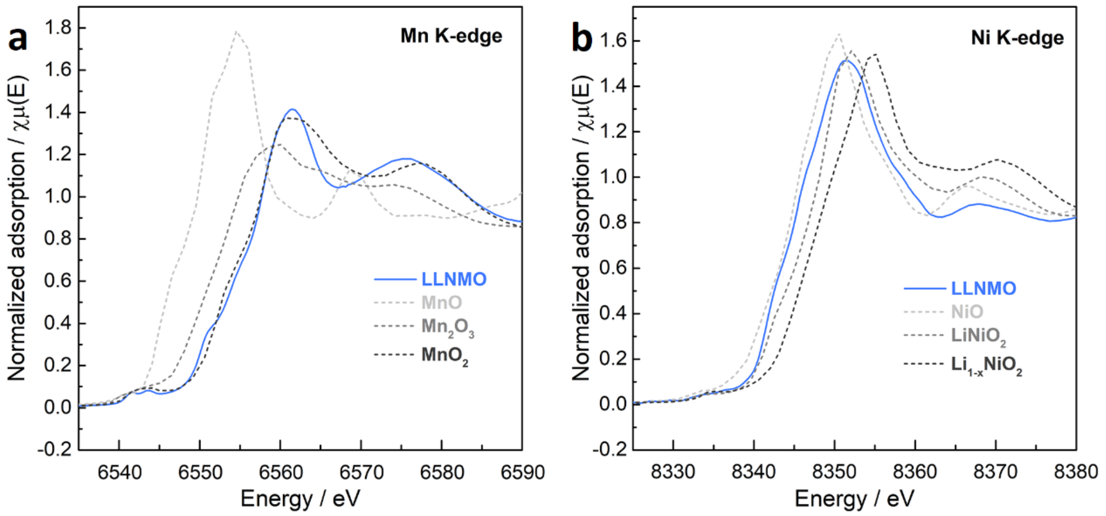

Figure S15. XAS results of pristine LLNMO. (a) Mn K-edge XAS results of MnO, Mn_2O_3, MnO_2, and the powder LLNMO. (b) Ni K-edge XAS results of NiO, $LiNiO_2$, $Li_{1-x}NiO_2$, and the powder LLNMO.

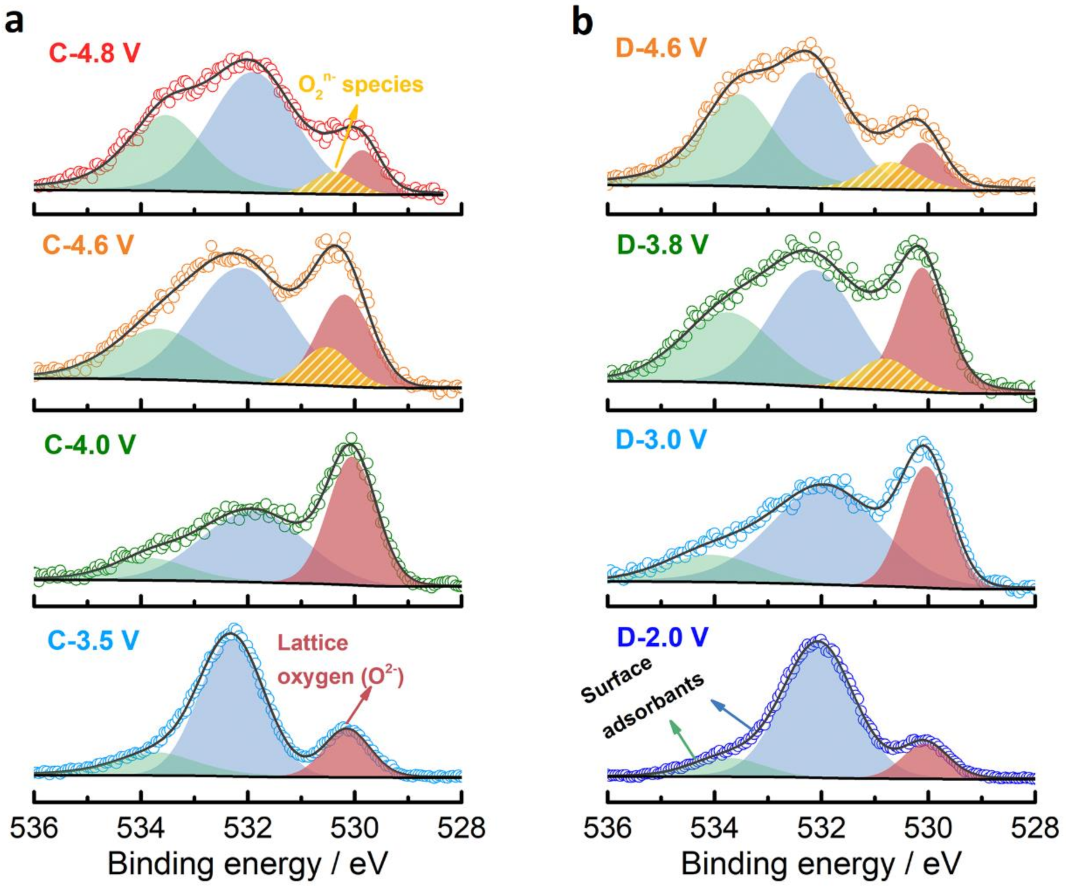

Figure S16. O 1s XPS spectra of LLNMO during (a) the 1st charge and (b) the 1st discharge. The peak position correction was applied based on the C 1s peak (284.8 eV)

The peaks at round 529.8 eV are intrinsic to lattice oxygen (O^{2-}), while the superimposed bands at around 532.5 eV are attributed to surface adsorbates and oxidized species. In the deeply charged states, the shoulders (i.e., yellow ones at around 530.5 eV) appear at binding energies higher than that of lattice oxygen (O^{2-}), indicating the formation of O_2^{n-} species ($0 \leq n < 4$).

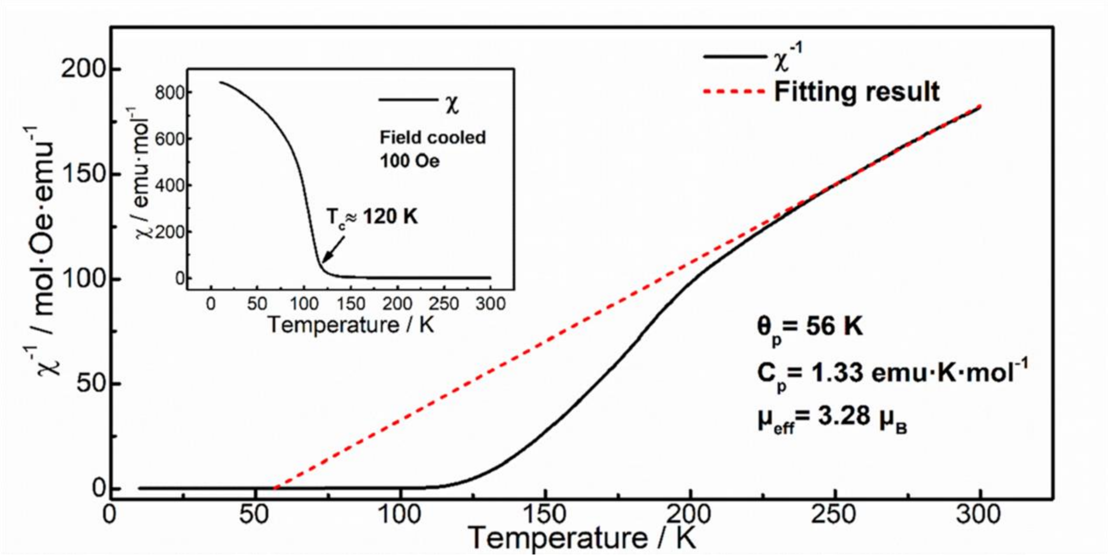

Figure S17. Magnetic susceptibility of the pristine LLNMO. The inset shows the field cooled temperature dependence of the magnetic susceptibility under the magnetic field of 100 Oe.

As shown in the magnetization curve under the magnetic field of 100 Oe (the inset), it presents a spontaneous strong magnetization at low temperature (10 K), which vanishes at the Curie temperature (T_c ≈ 120 K), identical to a ferromagnetic-to-paramagnetic transformation. In the high-temperature paramagnetic region (T > 200 K), the relationship between the magnetic susceptibility (χ) and temperature (T) follows the Curie-Weiss law:[21] $\chi=C_p$ i/$(T-\theta_p)$, where C_p is the Curie constant (emu·K/ mol) and θ_p is the Weiss temperature (K). The linear fit of the temperature dependence of the reciprocal magnetic susceptibility (χ^{-1}) in the paramagnetic region yields a Curie constant C_p=1.33 emu·K/ mol and a Weiss constant θ_p= 56 K. The positive value of θ_p indicates the dominance of ferromagnetic interactions, probably arising from intralayer super-exchange interactions mediated via the oxygen.[22]

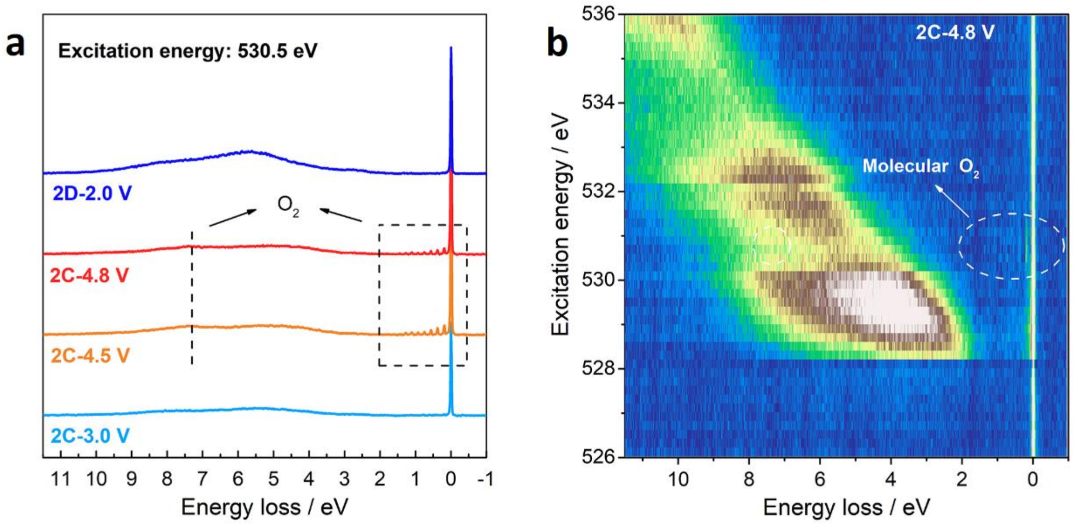

Figure S18. Bulk oxygen redox activities in the 2nd cycle. (a) HR-RIXS line scans obtained at the excitation energy of 530.5 eV. (b) The RIXS map of O K-edge at 2C-4.8 V. The molecular O_2-related features appear in charge and disappear after discharge, manifesting the reversibility of oxygen redox in the 2nd cycle.

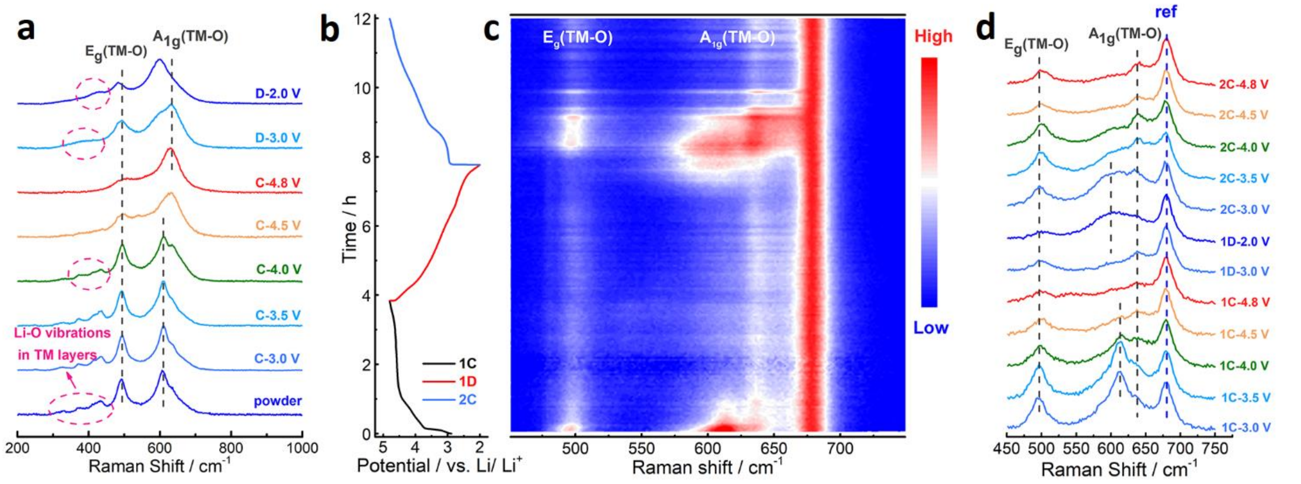

Figure S19. Local coordination evolutions of LLNMO upon de-/lithiation. (a) *Ex-situ* Raman spectra of the powder sample and LLNMO electrodes collected at different potentials during the 1st cycle. (b-d) *Operando* Raman spectra of LLNMO during the 1st cycle and the 2nd charge. (b) Voltage profiles of the *in-situ* cell at the current density of 50 mA/g within the potential window of 2.0 - 4.8 V vs Li/Li^+. Contour map and (d) selected Raman spectra at different states. All spectra were normalized by the reference peak at around 680 cm^{-1}, intrinsic to the optic window of the *in-situ* cell.

Raman spectroscopy, sensitive to short-range patterns, is an ideal tool to probe TM-O coordination within TMO_6 octahedra.[23] As shown in **Figure S19a**, in the region of 300-450 cm^{-1}, multiple peaks related to Li-O vibrations in TM layers gradually decay and disappear upon de-lithiation. Despite their recovery after lithium intercalation, those peaks become broader due to irreversible local arrangements in the 1st activation process. Correspondingly, the E_g peak at around 492 cm^{-1} also broadens in the deeply charged state (i.e., 4.8 V vs Li/Li^+) thanks to distortions of TMO_6 octahedra. In the region of 550-650 cm^{-1}, Raman peaks could be assigned to multiple symmetric TM-O vibrations parallel to the *c*-axis (A_{1g} modes), resulting from complex TM-O interactions in the bimetallic oxide. Specifically, for the pristine sample, the peak at around 606 cm^{-1} and the shoulder at 633 cm^{-1} are tentatively assigned to A_{1g} mode vibrations in $TM^{3+}O_6$ octahedra and $TM^{4+}O_6$ octahedra, respectively.[24] Upon charging to 4.5 V vs Li/Li^+, the A_{1g} peak at 606 cm^{-1} blue-shifts and overlaps with the shoulder to form a superimposed band at 633 cm^{-1}, resulting from cation oxidation and corresponding distortions of TMO_6 octahedra. When the electrode is charged to 4.8 V vs Li/Li^+, both E_g and A_{1g} peaks solely broaden without new peak formation, implying that local environments around oxygen remain stable. Upon discharging, the broad A_{1g} band splits at 3.0 V vs Li/Li^+ because of the partially reduction of TM ions. It finally red-shifts to 599 cm^{-1} at 2.0 V vs Li/Li^+, which echoes the proposed Mn^{4+} to Mn^{3+} reduction. *In-situ* Raman results also reveal irreversible peak broadening during the initial activation and reversible local structure evolutions thereafter (**Figure S19b-d**).

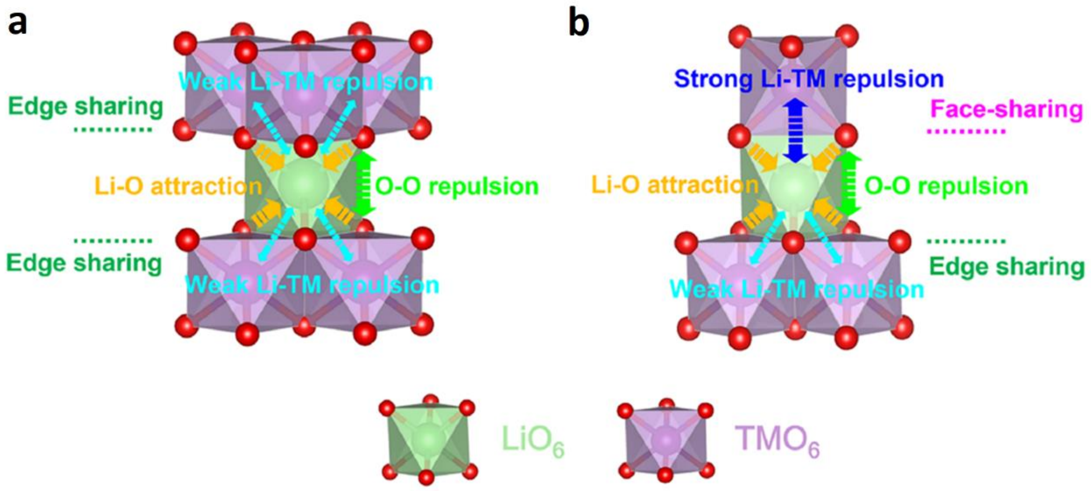

Figure S20. Schematic illustrations of interlayer interactions. (a) Interlayer interactions in the O3-type structure. A LiO_6 octahedron shares six edges with six TMO_6 octahedra on two sides along the *c*-axis. The Li^+-TM^{n+} repulsion is weak. (b) Interlayer interactions in the metastable LLNMO with face-sharing local patterns. A LiO_6 octahedron shares three edges with three TMO_6 octahedra on one side while shares a face with a TMO_6 octahedron on the other side. The Li^+-TM^{n+} repulsions at face-sharing sites are strong.

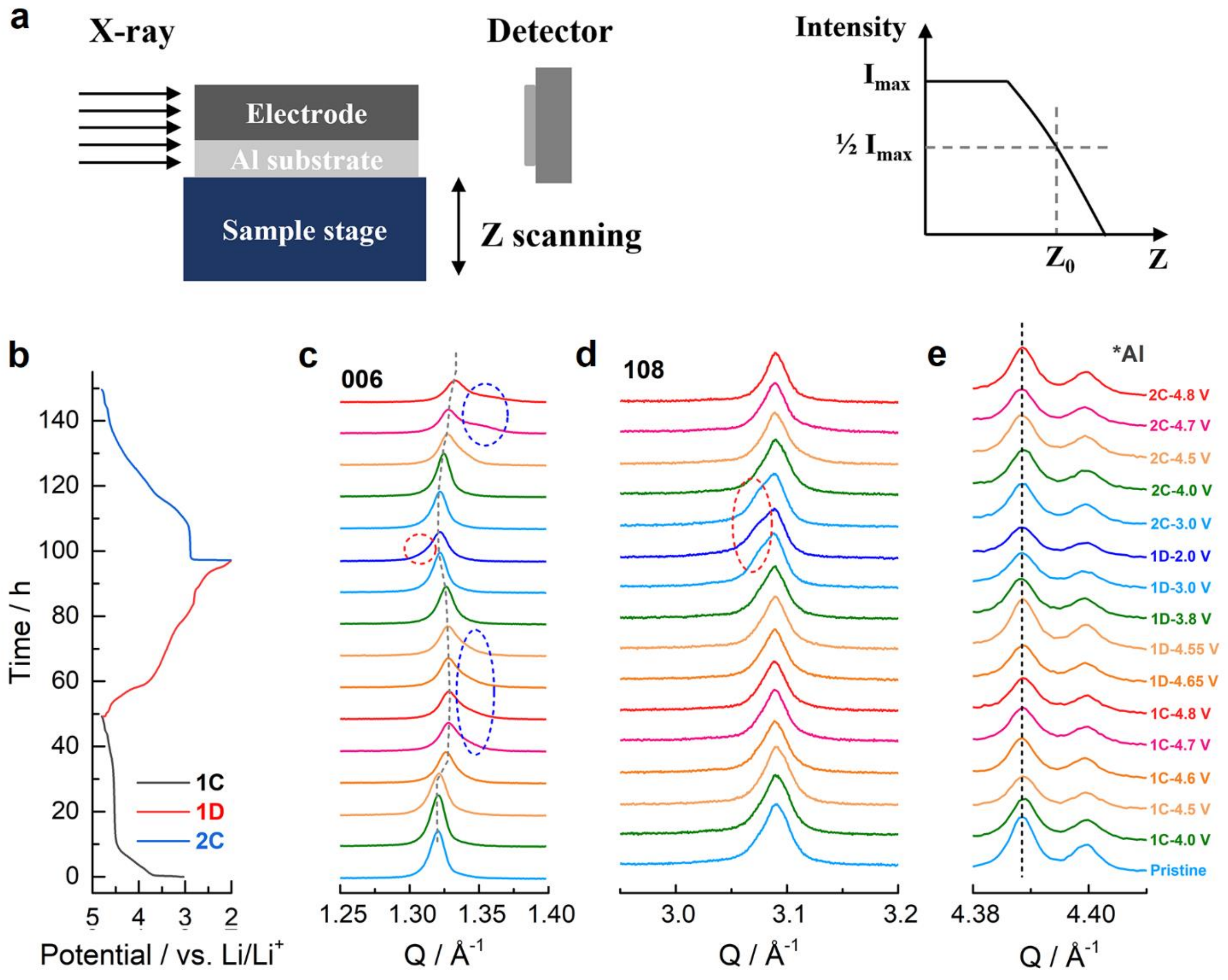

Figure S21. *Ex-situ* XRD results of the LLNMO. (a) Schematic illustration of the pre-test Z-calibration method. (b) Typical voltage profiles of the LLNMO electrode in the 1st cycle and the 2nd charge at a current density of 5 mA/g. The corresponding *ex-situ* XRD patterns (Cu Kα, λ=1.54 Å) of (c) the (006) peak, (d) the (018) peak and (e) the intrinsic peaks of the Al substrate at different states of charge and discharge.

To achieve precise Z calibration for reliable *ex-situ* XRD tests, an automatic height calibration process was performed before every single XRD test (denoted as the pre-test Z calibration method). Firstly, to avoid surface curvatures, all electrodes collected from cycled cells were flattened and put on a flat sample stage. Then, as illustrated in **Figure S21a**, the incident X-ray was fixed at the position in parallel with the flat sample surface (2θ = 0°). The sample stage was moved down to out of the X-ray pathway and the maximum intensity (I_{max}) was achieved on the detector. Upon moving up the sample stage, the position where the detected intensity was half of the maximum was regarded as the standard position Z_0. The following θ-2θ scanning was done without changing the height. After calibration, the intrinsic peaks of the Al substrate are at the same position for all samples (**Figure S21e**), proving the reliability of such a pre-test Z calibration method.

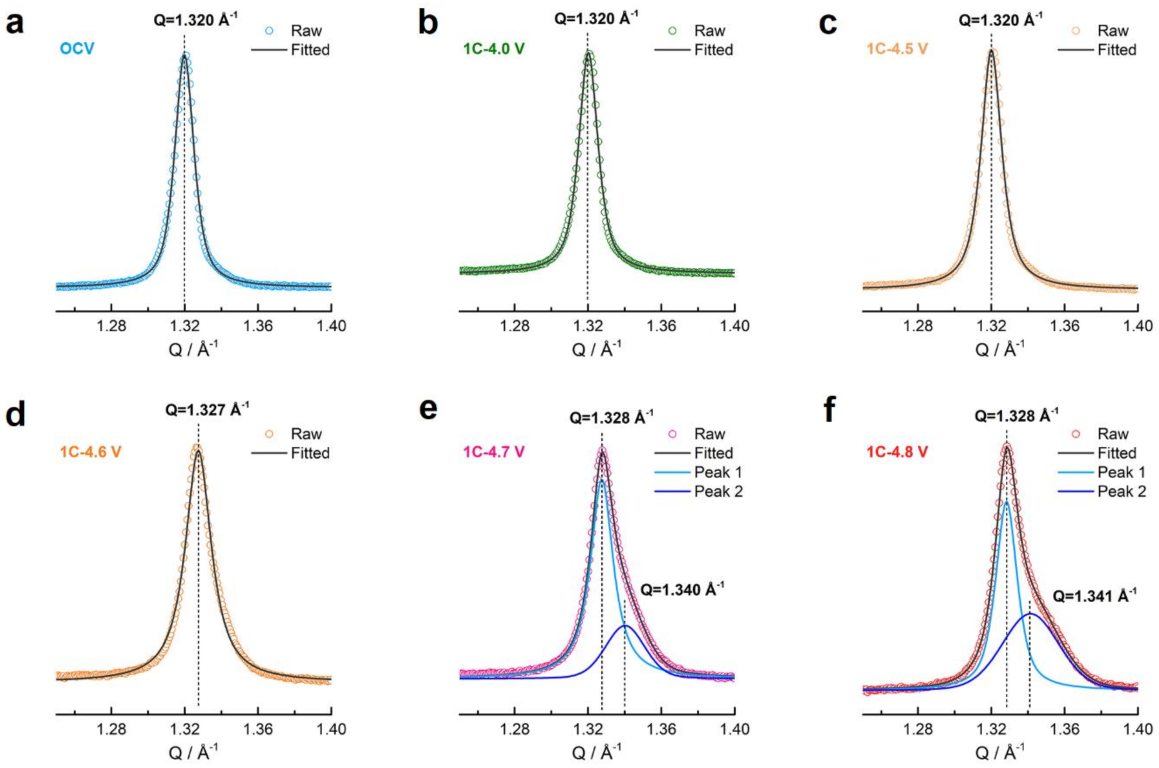

Figure S22. XRD patterns (Cu Kα, λ=1.54 Å) of the LLNMO electrodes collected at different states in the 1st charge. Fitted (006) peaks of the LLNMO electrodes at (a) OCV, (b) 1C-4.0 V, (c) 1C-4.5 V, (d) 1C-4.6 V, (e) 1C-4.7 V, and (f) 1C-4.8 V.

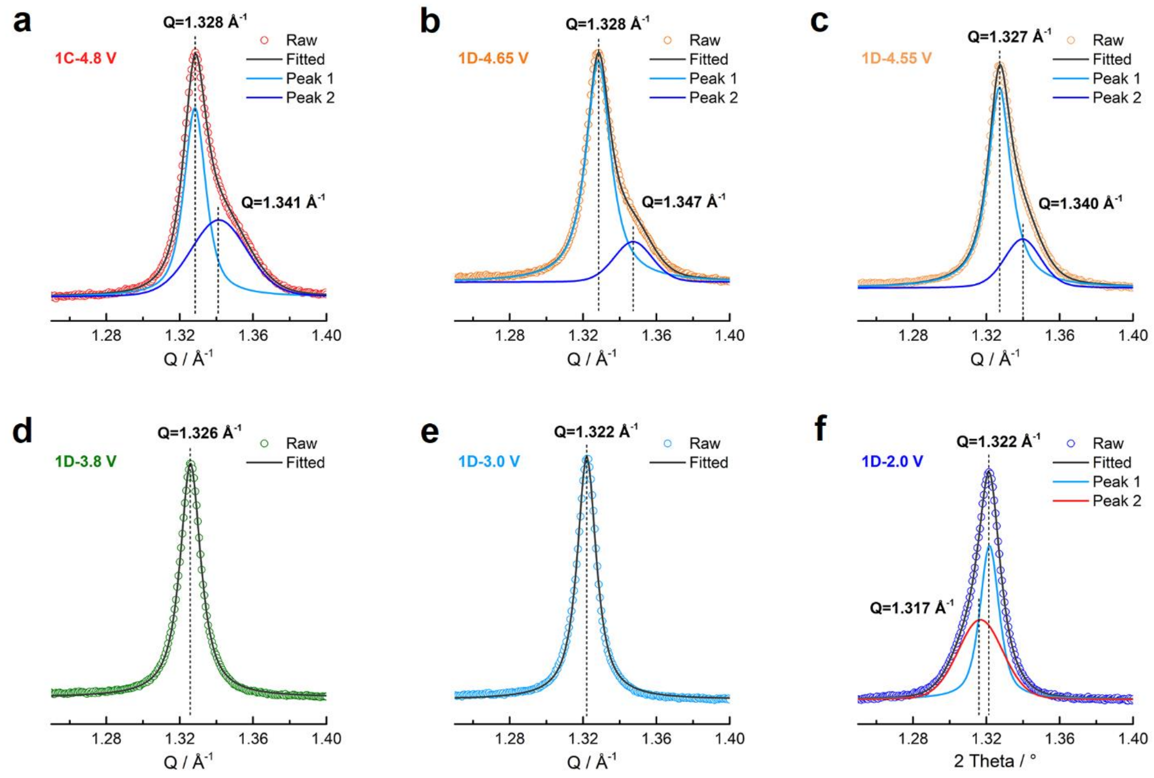

Figure S23. XRD patterns (Cu Kα, λ=1.54 Å) of the LLNMO electrodes collected at different states in the 1st discharge. Fitted (006) peaks of the LLNMO electrodes at (a) 1C-4.8 V, (b) 1D-4.65 V, (c) 1D-4.55 V,

(d) 1D-3.8 V, (e) 1D-3.0 V, and (f) 1D-2.0 V.

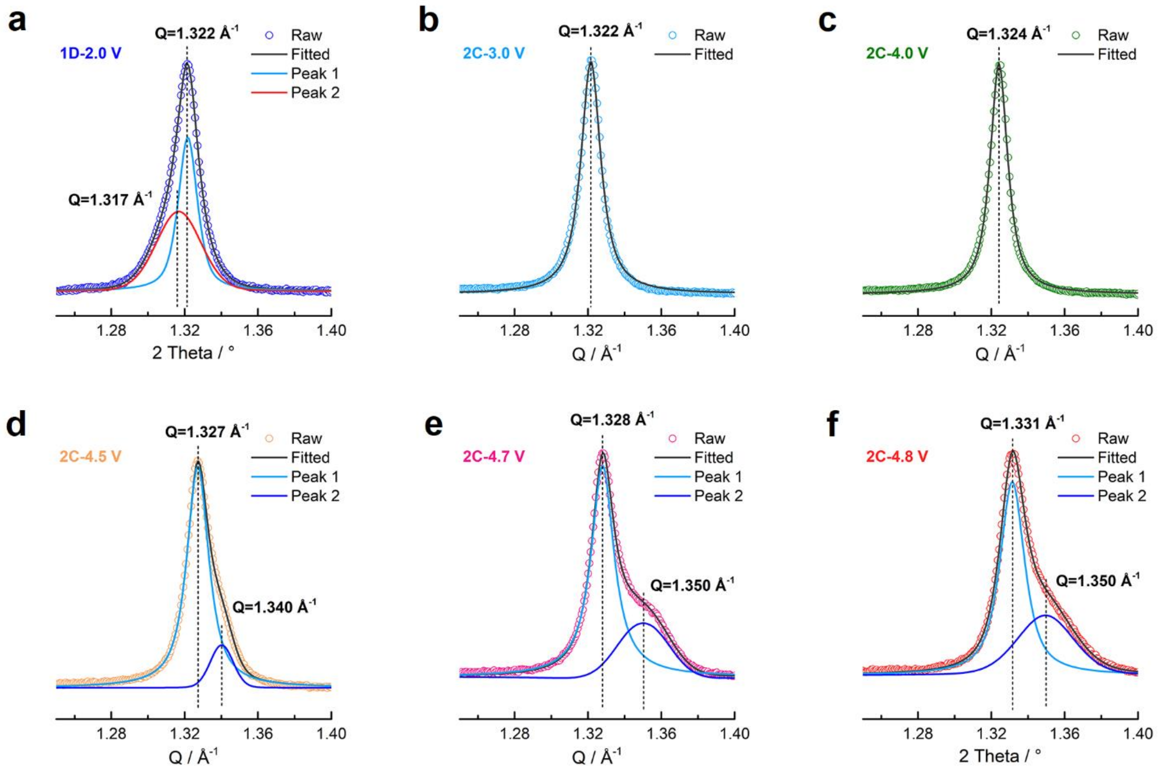

Figure S24. XRD patterns (Cu Kα, λ=1.54 Å) of the LLNMO electrodes collected at different states in the 2nd charge. Fitted (006) peaks of the LLNMO electrodes at (a) 1D-2.0 V, (b) 2C-3.0 V, (c) 2C-4.0 V, (d) 2C-4.5 V, (e) 2C-4.7 V, and (f) 2C-4.8 V.

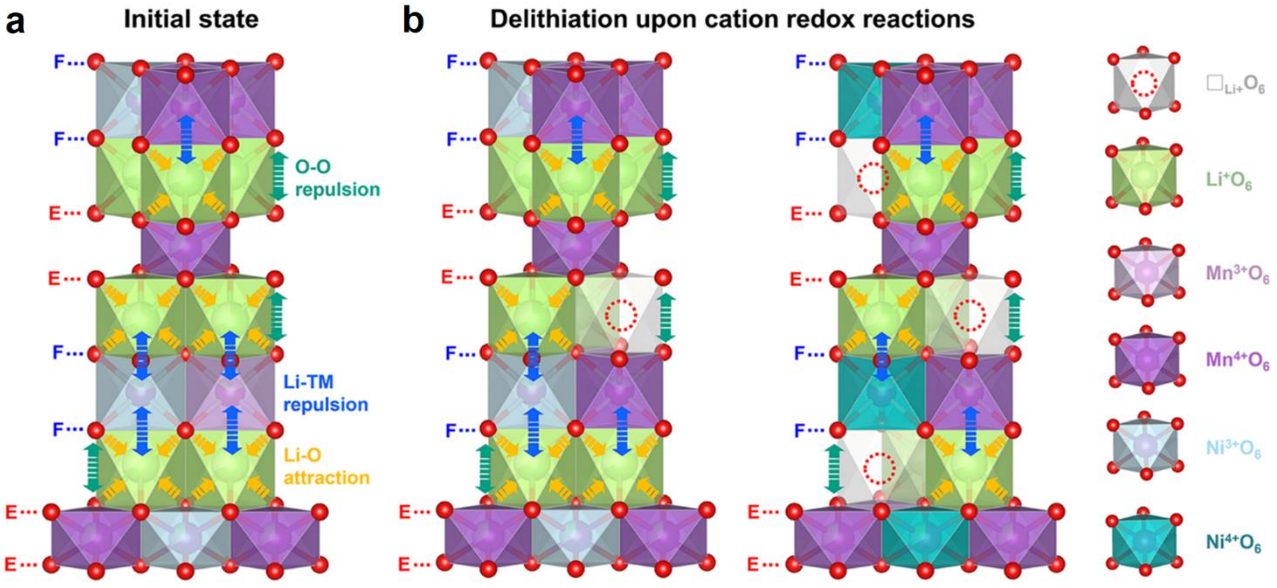

Figure S25. Evolutions of interlayer interactions. Schematic illustrations of the interlayer interactions (a) in the pristine LLNMO and (b) upon cation oxidation. E and F denotes edge-sharing and face-sharing sites, respectively.

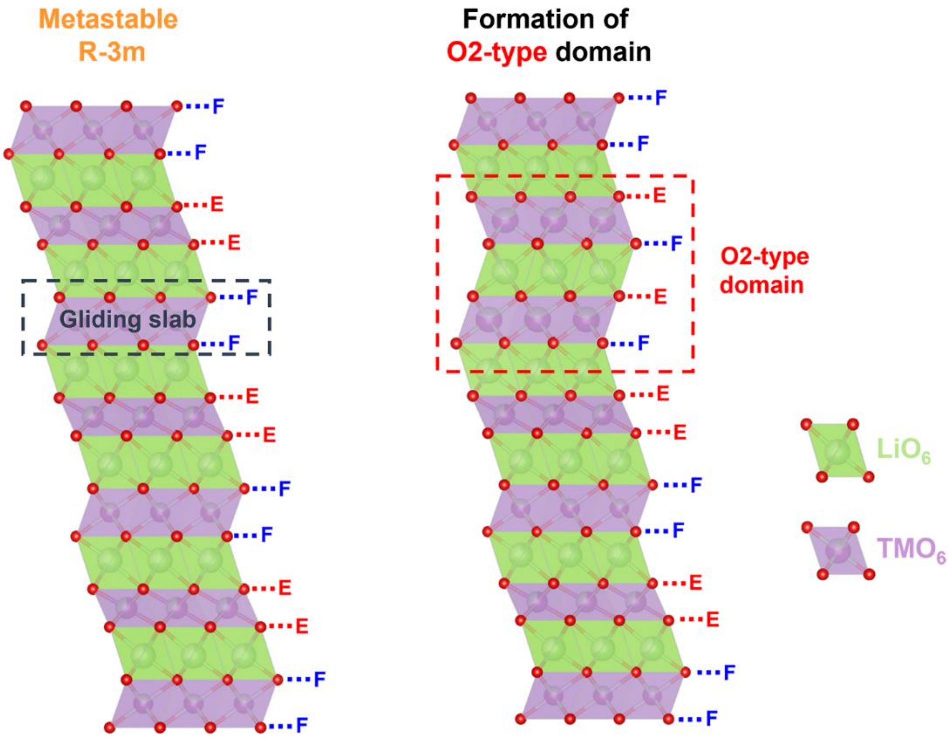

Figure S26. Schematic illustration of the formation of the O2-type domain in the deeply charged state. The TMO_2 slab sharing faces with adjacent LiO_2 slabs glide within the *ab*-plane. E and F denotes edge-sharing and face-sharing sites, respectively.

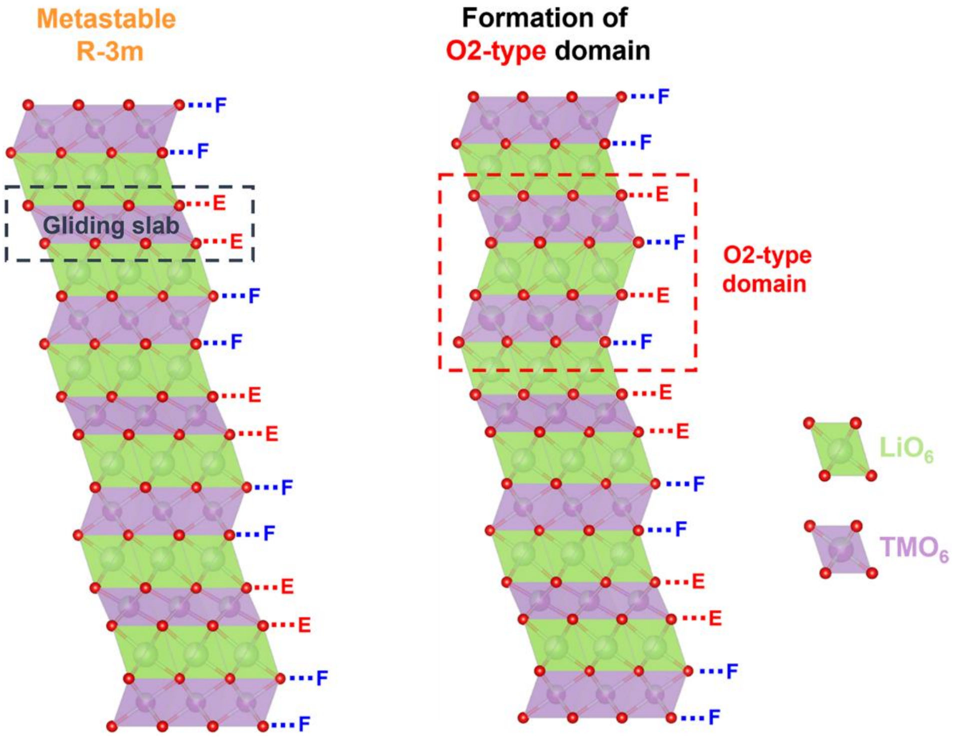

Figure S27. Schematic illustration of the formation of the O2-type domain in the deeply discharged state. The TMO_2 slab sharing edges with adjacent LiO_2 slabs glide within the *ab*-plane. E and F denotes edge-sharing and face-sharing sites, respectively.

Supplementary References

[1] B. H. Toby, *J. Appl. Cryst.* **2001**, *34*, 210-213.

[2] B. H. Toby, R. B. Von Dreele, *J. Appl. Cryst.* **2013**, *46*, 544-549.

[3] K.-J. Zhou, A. Walters, M. Garcia-Fernandez, T. Rice, M. Hand, A. Nag, J. Li, S. Agrestini, P. Garland, H. Wang, S. Alcock, I. Nistea, B. Nutter, N. Rubies, G. Knap, M. Gaughran, F. Yuan, P. Chang, J. Emmins, G. Howell, *J. Synchrotron Radiat.* **2022**, *29*, 563-580.

[5] M. Diaz-Lopez, G. L. Cutts, P. K. Allan, D. S. Keeble, A. Ross, W. Pralong, G. Spiekermann, P. A. Chater, *J. Synchrotron Radiat.* **2020**, 27, 1190-1199.

[5] Y. Wang, Y. Li, Z. Li, N. Qin, F. Wu, J. W. Makepeace, F. Zhang, P. K. Allan, Z. Lu, *ACS Energy Lett.* **2023**, *8*, 4888-4894.

[6] N. Yabuuchi, R. Hara, M. Kajiyama, K. Kubota, T. Ishigaki, A. Hoshikawa, S. Komaba, *Adv. Energy Mater.* **2014**, *4*, 1301453.

[7] Z. Yang, J. Zhong, J. Feng, J. Li, F. Kang, *Adv. Funct. Mater.* **2021**, *31*, 2103594.

[8] J. Feng, Y.-S. Jiang, F.-D. Yu, W. Ke, L.-F. Que, J.-G. Duh, Z.-B. Wang, *J. Energy Chem.* **2022**, *66*, 666-675.

[9] X. Cao, H. Li, Y. Qiao, M. Jia, H. Kitaura, J. Zhang, P. He, J. Cabana, H. Zhou, *Sci. Bull.* **2022**, *67*, 381-388.

[10] W. Hua, S. Wang, K. Wang, A. Missyul, Q. Fu, M. S. D. Darma, H. Li, V. Baran, L. Liu, C. Kübel, J. R. Binder, M. Knapp, H. Ehrenberg, S. Indris, *Chem. Mater.* **2021**, *33*, 5606-5617.

[11] D. Eum, B. Kim, S. J. Kim, H. Park, J. Wu, S.-P. Cho, G. Yoon, M. H. Lee, S.-K. Jung, W. Yang, W. M. Seong, K. Ku, O. Tamwattana, S. K. Park, I. Hwang, K. Kang, *Nat. Mater.* **2020**, *19*, 419-427.

[12] H. Liu, C. Zhao, Q. Qiu, B. Hu, F. Geng, J. Li, W. Tong, B. Hu, C. Li, *J. Phys. Chem. Lett.* **2021**, *12*, 8740-8748.

[13] X. Yang, T. Zhao, X. Zhai, J. Zhang, S. Wang, W. Hua, *Ind. Eng. Chem. Res.* **2024**, *63*, 4197-420.

[14] H.-Y. Jang, D. Eum, J. Cho, J. Lim, Y. Lee, J.-H. Song, H. Park, B. Kim, D.-H. Kim, S.-P. Cho, S. Jo, J. H. Heo, S. Lee, J. Lim, K. Kang, *Nat. Commun.* **2024**, *15*, 1288.

[15] Z. Yao, Y. Chen, C. Liu, H. Chen, S. Wu, D. Luo, Z. Lin, S. Zhang, *J. Energy Chem.* **2023**, *82*, 513-520.

[16] X. Cao, H. Li, Y. Qiao, P. He, Y. Qian, X. Yue, M. Jia, J. Cabana, H. Zhou, *Joule* **2022**, *6*, 1-14.

[17] X. Cao, H. Li, Y. Qiao, M. Jia, X. Li, J. Cabana, H. S. Zhou, *Adv. Mater.* **2021**, *33*, 2004280.

[18] K. Edström, M. Herstedt, D. P. Abraham, *J. Power Sources* **2006**, *153*, 380-384.

[19] S. Malmgren, K., Ciosek, M. Hahlin, T. Gustafsson, M. Gorgoi, H. Rensmo, K. Edström, *Electrochim. Acta* **2013**, *97*, 23-32.

[20] C. Cui, X. Fan, X. Zhou, J. Chen, Q. Wang, L. Ma, C. Yang, E. Hu, X.-Q. Yang, C. Wang, *J. Am. Chem. Soc.* **2020**, *142*, 8918-8927.

[21] C. M. Julien, A. Ait-Salah, A. Mauger, F. Gendron, *Ionics* **2006**, *12*, 21-32.

[22] D. Khomskii, M. V. Mostovoy, *J. Phys. A: Math. Gen.* **2003**, *36*, 9197-9207.

[23] Q. Wu, V. A. Maroni, D. J. Gosztola, D. J. Miller, D. W. Dees, W. Lu, *J. Electrochem. Soc.* **2015**, 162, A1255-A1264.

[24] P. Lanz, C. Villevieille, P. Novák, *Electrochim. Acta* **2013**, 109, 426-432.
